# Inventory optimization under tri phased demand with dual aging and controlled backlogging

**DOI:** 10.1016/j.isci.2026.115268

**Published:** 2026-03-06

**Authors:** E. Arunadevi, S. Umamaheswari

**Affiliations:** 1 Department of Mathematics, School of Advanced Sciences, Vellore Institute of Technology, Chennai 600127, India; 2 Department of Mathematics, School of Advanced Sciences, Vellore Institute of Technology, Chennai 600127, India

**Keywords:** applied sciences, agricultural science, agricultural engineering

## Abstract

The proposed study will create a unified inventory model that reduces the overall inventory cost and improves sustainability of seasonal and perishable goods. Seasonal and perishable inventories require superior models that are sensitive to the product attributes and fluctuating consumer demand. This article proposes a mathematical model in which both amelioration and deterioration phenomena are captured using a Weibull distribution and realistic product behavior is possible. The seasonal demand can be offered as a trapezoidal form of demand that varies with time and price, and a cubic holding cost form can be used to demonstrate the nonlinear rise in holding cost. The model also enables the backlogging of products to a certain degree in order to make shortages practical, and it includes investment in preservation to reduce the acceleration of the deterioration process and ensure quality of the product. Numerical examples and simulation studies under varying conditions of seasons confirm the model. The hybrid genetic algorithm (GA) and krill herd algorithm (KHA) optimization model proves to be very stable, computationally accurate with good convergence behavior, which justifies the practicability of the proposed model in the real world.

## Highlights

• Model adds amelioration and deterioration to control inventory sustainably
• Seasonal trapezoidal demand associates time effects, price effects, and sustainability results
• GA and KHA metaheuristics optimize replenishment, pricing and preservation strategies
• The quality and lessening spoilage is maintained by preservation investment and partial backlogging

## Graphical abstract

**Figure:**
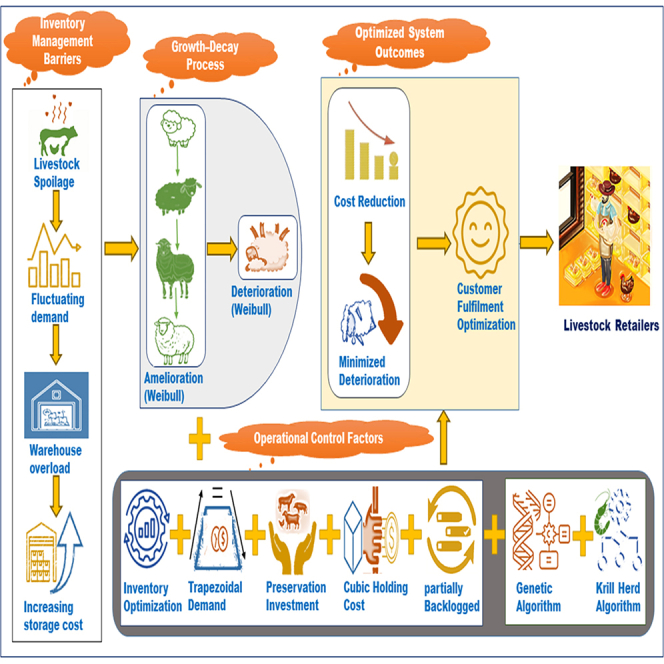

## Introduction

The biological improvement and inevitable decline are the two opposing forces in the livestock production that have to be reconciled in a dynamic valuation over time by inventory systems. As opposed to traditional inventory objects, animals are living organisms that are in constant flux, acquiring weight and market value through amelioration and at the same time losing it through old age, illness, or weather. Inventory models that use traditional approaches not usually take into consideration this dual behavior, which restricts their application in livestock and agricultural supply chains. To deal with this complexity, Hwang^1^^,^^2^ originally proposed the concept of amelioration and subsequently applied it to deterioration as the basis of more biological responsive models. This framework was extended by Arunadevi and Umamaheswari^3^ using Weibull distributions, which provides an opportunity to modulate changes in quality with time and prices, and in particular, in livestock systems. Weibull function shape and scale are estimated using numerical calibration against historical data of livestock growth and decay, and they determine the rate, by which a product value increases or decreases with time. The shape parameter is positive and bigger, which indicates quick amelioration or deterioration, which is a realistic tool to represent the biological variability.

The demand in the real life livestock operation is seasonal because of breeding season, festivals, climatic change, and consumer trends. There is a trapezoidal (tri-phased) demand function which is followed to reflect this variability and which phases the planning horizon into increasing, steady, and declining phases of demand. It is used instead of sinusoidal representation, exponential representation, or polynomial representation, since it better reflects the sharp and sharp turnover frequently observed on actual farm markets, as demand surges suddenly during party seasons, or demand falls precipitously when harvest is over, without the blurring effect of sinusoidal or exponential curves. This is the approach that was tested and proved right by Cheng et al.^4^ and offers a realistic demand structure applicable to perishable or seasonal goods. In addition, the cost of livestock maintenance is nonlinear holding costs, which are affected by factors like feeding, labor, space limitation and disease risk. A cubic holding cost function is added to capture these operational realities as it has been in the work of Thirugnanasambandam and Sivan.^5^ Such a nonlinear cost hypothesis is reflective of actual behavior in the real world where the marginal cost of keeping more animal increases faster at higher stock densities because space is finite, more animals are prone to disease and competition over resources. Empirical evidence of livestock farms supports the hypothesis that marginal cost of maintenance increases with time and size of the herd and justifies replacing a linear or quadratic form with a cubic formulation. Moreover, the limited usability life of livestock products is also taken care of in the manner of Umamaheswari et al.^6^ such that the model perfectly incorporates the biological, seasonal, and economic relationships.

Also, deterioration can be reduced by preservation investment, such as the use of vaccination, optimal nutrition, and improving shelter. Hsu et al.^7^ and Dye and Hsieh^8^ confirm in their previous studies that inventory performance is improved by investment in preservation strategies. In the meantime, partial backlogging characterizes best shortage behavior in livestock markets, as not all unmet demand is postponed, some customers will find alternatives, or cancel their orders altogether. This is especially applicable to the cases of trade credit policies as presented in the writings by Jayashri and Umamaheswari,^9^^,^^10^ who also modeled perishables whose supply chain relationships were based on credit.

Even though the present research has contributed to the field of livestock inventory, most of the studies have considered the biological changes, the demand change with the season, the cubic holding costs, preservation procedures, and thirdly, shortage among others in isolated or minimal combinations. To reduce this gap, the current study constructs an extensive livestock inventory model, which incorporates all these important factors into one biologically based and computationally optimized model. Besides, the research expands the idea of sustainability to include maintenance, reduction of waste, efficient use of resources, and reduced overstocking. This system based approach indirectly decreases feed intake and energy consumption and lessens environmental influence hence extending sustainability to both environmental and operational scales. This has led to no studies that have in a single framework of study taken into consideration both the Weibull-based amelioration and deterioration, three-phase demand, cubic holding cost, preservation investment, backlogging in parts, and hybrid metaheuristic optimization. Based on this, the research questions to be answered in the study are as follows:

• How does the joint modeling of amelioration and deterioration (via the Weibull distribution) effect optimal replenishment and preservation decisions for livestock inventories?
• To what extent do tri-phased (trapezoidal) seasonal demand patterns and price sensitivity alter inventory and preservation policies?
• How do cubic holding costs and partial backlogging influence total inventory cost and service-level trade-offs in livestock systems?
• Can a hybrid optimization scheme (genetic algorithm [GA] and krill herd algorithm [KHA]) reliably identify near-optimal policies and improve computational performance for this complex model?

The optimization framework adopted in this study is presented in the graphical abstract, providing a structural overview of the model development and solution process. Although the different parameters of inventory control have been addressed separately, there are no integrated models to capture the combined impacts of the life cycle dynamics of animals, market fluctuations and operational limitations. To achieve a solution to this methodological gap, a hybrid livestock inventory framework is provided in the current study in which the following are recorded:

• Simultaneous amelioration and deterioration using the Weibull distribution
• Trapezoidal demand functions that vary with both time and price
• Cubic holding cost structures reflecting real-world resource intensities
• Partial backlogging to reflect realistic customer waiting behavior
• Investment in preservation to delay quality loss
• Optimization through GA and KHA for solving complex nonlinear problems.

Validation of the proposed model is carried out using numerical experiments and simulation-based sensitivity analyses. The performance of this model is compared to the traditional inventory model that does not have either the preservation or cubic cost aspect to indicate that it is superior in reducing the overall cost and improving the sustainability effect.

### Novelties

The suggested model sets a new standard of biologically motivated optimization of the inventory by incorporating real-life product behavior, realistic market readjustment, and sustainability into a single framework. Contrary to the past works which have focused on these elements individually, this article integrates amelioration, deterioration, preservation, and shortage decisions into a single decision system that is adaptable to complex, time-constrained environments. The hybrid GA and KHA algorithmic engine has high convergence speed, stability and accuracy, and is found to have high reliability in diverse seasonal conditions compared to the traditional metaheuristics. In addition to computed benefits, the model will display real benefits operationally over minimizing spoilage, reducing holding costs, and increasing levels of service at the same time. It makes the inventory management less of a fixed-cost control operation and more of an adaptive adaptation to the market through optimization. Such a combination of analytical depth, computational power, and sustainability orientation makes the proposed model one of the most thorough and practically relevant approaches to research related to inventory in modern times.

### Literature review

Inventory modeling of perishable and ameliorating products has undergone an incredible development, incorporating practicalities of complexities like amelioration, deterioration, preservation investment , trade credit, sustainability and financial strategies. The Arunadevi et al.^11^ model of livestock inventory considers Weibull based amelioration and deterioration and it combines the strategies of the preservation investment of livestock; the strategy of rebate is both static and dynamic. The application of GA and bat algorithm (BA) made it clear that rebate and preservation are synergistic to improve profit and sustainability. On the same note, Hatibaruah and Saha^12^ investigated the simultaneous amelioration and deterioration under the influence of stock, advertising, preservation investment and trade credit and found out that the cost and demand uncertainties have a significant impact on profitability. Jayashri and Umamaheswari^13^ proposed a livestock model based on trade and credit in the livestock industry in developing fast-growing products like broilers, in which the financial results were improved by optimization of the replenishment time and credit period.

The recent past has been full of research on livestock and sustainable production systems. In a comparison between the carbon tax (CT) and the cap-and-trade (C&T) systems, Khan et al.^14^ developed an optimization model to estimate investment to reduce emission of livestock farms using non-linear holding cost and power demand. Their findings showed that the C&T policy creates a higher degree of flexibility and cost-effectiveness, which encourages sustainability in the long term. Khan et al.^15^ in another study developed a livestock inventory model which combined pricing and prepayment with carbon emission policies (CAP, CAT, CAO, and CT) and power demand and demonstrated that minimization of cost related to emissions enhances the profit and ecological performance. Beyond that, it has been further elaborated by Khan et al.^16^ that a hybrid necessary to grow item acquisition of incremental quantity discounts and tax laws was created using a comparison differentiation hybrid optimization technique, and determination of the improvement of CT integration to the economic environmental equilibrium. Similarly, Gharaei and Almehdawe^17^ suggested a sustainable EOQ framework of expanding agricultural products with the inclusion of GHG emission expenses on manure, fermentation and transportation and used a combined HHO-GWO metaheuristic to attain low inventory expense and optimum sustainability outcomes.

A number of studies have highlighted the incorporation of sustainability in economic choices. Pervin et al.^18^ formulated integrated vendor-buyer model that includes remanufacturing of the returned goods to match the environmental, social and economic objectives. Their results substantiated the fact that remanufacturing enhances sustainability and profitability, through cutback in carbon emissions. Equally, Mukherjee et al.^19^ went a step further and applied the classical economic production quantity model to include price-sensitive and green-sensitive demand, taxation on carbon emission, and preservation technology and showed that the role of green investment and taxation on carbon emission in the context of uncertainty is significant to profitability. Barman et al.^20^ also suggested a Pythagorean fuzzy EOQ model, which incorporated hybrid price-stock-dependent demand, preservation technology, and advance payment scheme, and demonstrated that fuzzy-based models are better in accuracy making profits and uncertainty of deterioration, lead time, and cost parameters.

To cover the financial aspect, Kumar Ghosh et al.^21^ worked on a two-layer supply chain model that combines the hybrid payment of retailers and the trade credit of suppliers in the case of stochastic demand and demonstrate that partial prepayment and strategic borrowing enhances the profitability and the financial soundness. On the same note, Mallick et al.^22^ developed a perishable goods-based supply chain model with a two-level supply chain taking into consideration both fuzzy lead time and shortages and established that a fully backlogged shortage system is more profitable. Manna and Bhunia^23^ introduced a sustainable production model in which the demand is determined by the selling price and energy consumption, and it is noted that the energy-efficient manufacturing investment is better at the environmental and economic performance.

In the framework of deterioration-amelioration, Patra et al.^24^ formed an inventory model that is power-pattern demand, trade credit, and preservation investment and demonstrated that preservation investing decreases deterioration and enhances profit, particularly in fuzzy environments. Nayak et al.^25^ developed the model of a retailer whereby Weibull amelioration and constant deterioration are used, which reduces the overall cost but solves the issue of fuzzy cost imprecision. This was further extended by Patra et al.^26^ who added the concept of partial backlogging and established that fuzzy techniques provide more realistic and profitable decisions. Kumar and Paikray^27^ formed a model of deteriorating items that are operated in the trapezoidal demand with utterly backlogged shortages that made use of trapezoidal fuzzy numbers and concluded that fuzzy systems enhance flexibility and decision-making in uncertain markets. In a similar vein, Kumar et al.^28^ introduced a Weibull-deteriorating inventory model including positive lead time and various payment schemes to conclude that flexible payment schemes and lead-time factors play a major role in enhancing profitability and bolstering managerial decision-making. Varied on these investigations, Patra et al.^29^ proposed a model whereby there is a pattern of power-demand with learning effect in the context of backlogged shortages with a marked reduction in the costs. In a similar contribution, Patra et al.^30^ have introduced an inventory model, which combines constant deterioration, power pattern demand, allowable shortages, partial backlogging, and fuzzy cost imprecision and they provide more information on the cost minimization under complex operations.

The combination of demand dynamics and preservation investments is supported by further research. Chakrabarty et al.^31^ examined the production of greenhouse flowers and plants under trapezoidal demand and stage mortality, and found out that it is the emission policy that directly influences the cost of operation and the choice of stocks. Chang et al.^32^ analyzed perishable products that are subject to uncertain demand with a partial backlog and flexible payment demonstrating the efficiency enhancement by financial flexibility. Cheng and Wang^33^ and Cheng et al.^4^ were the first to use trapezoidal demand functions, which they found useful in the modeling of life cycle-based demand variations of deterioration and credit. Kaushik^34^ determined that preservation at the stage of mid cycles has the best profit maximization, and Mishra^35^ confirmed that preservation investments decrease the deterioration and increase sales. Thirugnanasambandam and Sivan^5^ integrated quadratic demand and cubic holding costs, finding that ordering and holding expenses account for the majority of total costs. Likewise, Vandana and Srivastava^36^ modeled time-dependent amelioration in livestock systems during seasonal cycles, while Xu et al.^37^ and Zhao^38^ confirmed that trapezoidal and Weibull-based demand functions better capture real-world deterioration and replenishment dynamics. Patra et al.^30^ presented a comprehensive inventory model for deteriorating items under a power-demand pattern with a learning effect and backlogged shortages, showing that learning significantly reduces total cost and enhances replenishment efficiency. Kumar et al.^28^ developed realistic inventory model in the form of Weibull-deteriorating items with positive lead time and multiple payment regimes and found out that flexible payment and lead-time schemes contribute to higher profitability and robustness of decisions.

The optimization algorithms have found their way into the mainstream in the resolution of complex, nonlinear inventory systems. To handle the interval uncertainty, quantum-behaved PSO variants were used by Mondal et al.^39^ Arunadevi et al.^11^ utilized GA and BA to optimize price, replenishment, and inventory levels for livestock systems. Mandal et al.^40^ developed the tournament teaching-learning-based optimization (TTLBO) algorithm, which surpassed existing metaheuristics in speed and quality, proving its robustness for nonlinear cost optimization. Together, these studies and algorithms highlight that metaheuristic and fuzzy techniques provide superior adaptability for optimizing multi-variable and uncertainty-driven livestock systems.

From the overview presented in Table 1, it is evident that the existing literature demonstrates substantial progress in modeling amelioration, deterioration, sustainability, and financial interactions in perishable and livestock-based systems. However, most prior studies have analyzed these aspects separately rather than in an integrated manner, and only a few have jointly considered Weibull-based amelioration and deterioration, trapezoidal time and price-dependent demand, preservation investment, and cubic holding cost within a unified framework. Furthermore, partial backlogging and metaheuristic optimization remain underexplored in livestock-oriented inventory systems. Therefore, the present study addresses these research gaps by proposing an integrated, sustainability-oriented livestock inventory model that unifies these critical elements and employs GA and KHA for global cost optimization. This integrated framework is better in efficiency of the economy as well as environmental sustainability as a decision-support tool which can be practically implemented to find optimal livestock inventory management under practical complexities.

**Table 1:** Formalization of existing literature in identification of the incorporation of product amelioration, deterioration behavior, demand variability, preservation, non-linear holding cost, backlogging policy, and optimization techniques in inventory modeling models

Author(s)	Amelioration	Deterioration	Trapezoidal type demand	Preservation	Cubic holding cost	Partial backlogging	Metaheuristic optimization
Hwang^1^	Weibull	–	–	**–**	**–**	**–**	**–**
Thirugnanasambandam and Sivan^5^	–	Constant	–	–	Yes	–	–
Zhao^38^	–	Weibull	Time dependent	–	–	Yes	–
Xu et al.^37^	–	Constant	Time dependent	–	–	Yes	–
Chakrabarty et al.^31^	–	–	Time dependent	–	–	Yes	–
Kaushik^34^	–	Constant	Time and price dependent	Yes	–	Yes	–
Jayashri and Umamaheswari^13^	Weibull	Constant	–	–	–	–	GA and PSO
Hatibaruah and Saha^12^	Weibull	Weibull	–	Yes	–	–	–
Arunadevi and Umamaheswari^41^	Weibull	Constant	–	Yes	–	Yes	–
Vandana and Srivastava^36^	Weibull	Weibull	Time dependent	–	–	–	–
Supakar et al.^42^	–	Constant	–	Yes	–	–	Artificial bee colony
Mandal et al.^40^	–	Weibull	–	–	–	Yes	TTLBO
Formulated Model	Weibull	Weibull	Time and price dependent	Yes	Yes	Yes	GA and KHA

Although individual elements like amelioration, deterioration, preservation investment, trapezoidal demand patterns, nonlinear holding costs, trade credit, partial backlogging and others have been studied separately, they have been, in most cases, considered individually. Consequently, the available models have very minimal applications to the livestock and perishable inventory settings where growth behavior, rising costs and seasonal demand tend to be in coexistence. The present research addresses this gap in the research methodology by uniting Weibull-related amelioration and deterioration, demand, which can be price-dependent and time-dependent, trapezoidal, cubic holding cost, preservation, and partial backlogging within a single methodology. This is not answered in the previous literature, which makes the proposed model innovative and unique with respect to its relevance.

### Motivation, research gap, and contribution

#### Motivation

The livestock and perishable inventory systems present unique problems since the products of interest are biological (they grow [amelioration] and at the same time decay, become diseased or corrupted by the very environment). These biological dualities are very critical in the processes of replenishment, valuation, and sustainability of operations in real-world. Nevertheless, the majority of classical models of inventory treat these effects in isolation which restricts their use in industries. The recent studies conducted (Arunadevi and Umamaheswari^3^^,^^41^; Chakrabarty et al.^31^; and Hatibaruah and Saha^12^) have developed structures of sustainability orientation by incorporating preservation investments, environmental constraints, and demand variability. However, these models mostly focus on deterioration-preservation trade-off or the policy-based sustainability, and fail to explain the biological interdependence between amelioration and deterioration. Similarly, optimization models of trapezoidal-like demand (Xu et al.^37^) or the partial backlog usually use a linear cost of holding, which ignores the nonlinear increase in cost over time (e.g., cost of feeding, housing, and care), which is common in livestock systems. Driven by these inefficiencies, this article comes up with a more biologically plausible sustainable inventory model based on the non-linear, stochastic, and environmentally sensitive nature of the livestock processes in the real world.

#### Research gap

According to the literature review, a number of critical research gaps and gaps in integrating research has been stated.

• **Integration gap:** other authors have discussed amelioration and deterioration and preservation as separate aspects (Vandana and Srivastava^36^; Zhao^38^; Hatibaruah and Saha^12^; and Arunadevi and Umamaheswari^3^^,^^41^). However, an integrated formulation that simultaneously captures amelioration, deterioration interactions, trapezoidal time and price-dependent demand, and preservation investment is still missing.
• **Cost-structure gap:** most existing models assume linear or constant holding costs, which oversimplify real-world operations. Livestock inventory involves nonlinear (cubic) holding costs that reflect escalating expenditures for feed, healthcare, and maintenance, as observed in practical agricultural systems. Few studies have incorporated such nonlinear cost dynamics into sustainable inventory formulations.
• **Sustainability gap:** although some recent works (Arunadevi and Umamaheswari^41^; Chakrabarty et al.^31^) have integrated environmental and sustainability considerations, they do not adequately represent biological sustainability, the synergy between growth enhancement (amelioration) and deterioration control. The absence of a biologically grounded sustainability mechanism limits its industrial applicability.
• **Optimization gap:** traditional optimization approaches (e.g., single-objective or single-algorithm models) cannot effectively manage the nonlinear, multi-objective, and stochastic nature of biologically dynamic systems. None, because other metaheuristics, such as GA (Holland^43^) and KHA (Gandomi and Alavi^44^), have been applied singly, the benefits of hybrid metaheuristics in terms of exploration and exploitation have not been fully explored in the context of sustainable inventory optimization.
• **Real-world relevance gap:** a significant number of theoretical models have been insulated by the realities in the industrial world, particularly in the livestock and perishable products industries, where biological processes, cost-escalating, and partial backlogging are the main consideration in decision-making. The gap that exists in this study is filled by the development of a biologically viable and economically sound model.

Overall, this study demonstrates a significant deficiency in a single, sustainability-focused, and biologically realistic model of inventory that would incorporate amelioration, deterioration, preservation expenditure, nonlinear (cubic) cost increase, and trapezoidal demand with part backlogging.

#### Contributions

In order to address the limitations identified and align with the objectives of the research, the proposed study contributes to them in the following major ways.

• **Integrated nonlinear sustainable model:** the overall inventory model is developed to ameliorate and deteriorate items with the trapezoidal time and price-dependent demands. The model also integrates Weibull-distributed biological processes, cubic holding cost, preservation investment and partial backlogging providing realistic model of livestock and agricultural systems.
• **Formulation of biological sustainability:** presents a new biological sustainability paradigm involving the balance of preservation expenditure, quality enhancement, and environmental efficiency, between biological and environmental sustainability dimensions.
• **Advanced hybrid optimization:** the problem is a nonlinear and stochastic and multi-objective problem requiring a hybrid GA and KHA, and hence, providing strong convergence and high performance in comparison to single-method optimization.
• **Relevance to the industry:** the model can be directly applied to an agro-based industry like livestock where biological growth, cost escalation, and environmental stewardship co-exist, satisfying.

To conclude, the suggested study is driven by the practical biological and sustainability problems, bridges definite methodological and empirical research gaps, adds an integrative, decision-supporting model based on optimism, and contributes to the academic theory and commercial practice of sustainable inventory management.

### Methodology

This study constructs an integrated inventory model in the livestock systems, which considers a combination of both value additions and depreciations by using a Weibull distribution to estimate time-varying quality variations. To reflect the real-life consumer behavior, demand is defined as a trapezoidal form with seasonal and price driven variations based on the planning horizon into increasing, steady and declining periods. In order to model the increasing resource demand to keep livestock alive, cubic holding cost function is used to model the nonlinear increase in cost of keeping livestock including feeding, labor and shelter costs. The model also includes partial backlogging to reflect the customer reaction to shortages to reflect the fact that not all elements of unmet demand translate to postponed fulfillment, but lost sales. The decision variables such as preservation investments such as vaccination and optimized care are incorporated to curtail deterioration and increase the usability of products.

The goal is to reduce overall inventory cost through maximizing the replenishment quantity, preservation effort and cycle time. GA is based upon principles of selection, crossover and mutation to evolve a population of solutions, whereas KHA is based upon the herding behavior of individual krill, where movement is directed by induced motion, foraging motion, and random diffusion to global optima. Both the algorithms are designed mathematically in order to maintain the exploration and exploitation to converge toward near-optimal solutions of nonlinear and multidimensional problems.

The methodology of the solution is arranged like:

• Model parameters (demand, holding cost, preservation, and backlogging) are used to define the average total cost function.
• Set up GA and KHA algorithms with random feasible values of decision variables.
• Measure the fitness of every candidate solution based on the average total cost objective.
• Repeat solutions with GA operators (selection, crossover and mutation) and KHA operations (induced movement, foraging, and random motion).
• Stop the process as soon as convergence is achieved or some significant progress is perceived.

**Figure 1:**
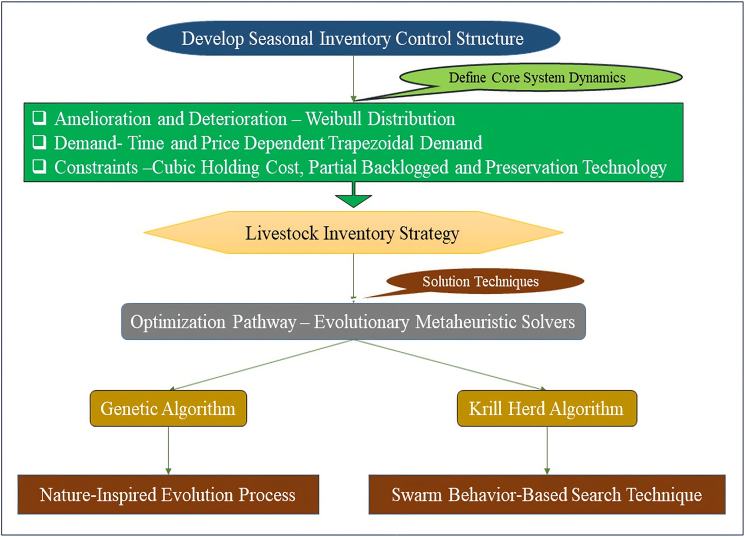
Proposed methodology for livestock inventory optimization using Weibull life cycle modeling and metaheuristic algorithms

The model is applicable with a great number of numerical examples that confirm the model as useful both under different demand and operational conditions as well as under biologically viable conditions that would support cost-effective operations to make inventory decisions, which would be practically applicable to livestock inventory management, as described in Figure 1.

### Notations and assumptions

#### Notations

*ψ*- Optimum order quantity whereby the average total cost is minimized.
*D* (*P*,*t*)- Trapezoidal demand function, which is related to the time *t* and selling price *P*.
*T*- Inventory cycle (i.e., fixed cycle time).
*R*_1_- The first step in the cycle where demand is not recorded.
*t*_1_- The particular time at which the inventory in the store will run out to zero.
*ω*_1_- The moment of time when the inventory of the first replenishment source runs out.
*ω*_2_- This is the time at which the inventory of the second source of replenishment is exhausted.
*g*_1_- Unit cost of ameliorating (improving) inventory items.
*g*_2_- Unit cost of deteriorating (decay) inventory items.
*g*_3_- Unit cost which is a result of system shortages.
*g*_4_- Unit cost of penalty incurred on a lost sale.
*αβ*- The cubic holding cost per unit of inventory, which includes linear and nonlinear elements of time.
*ξ*- Preservation investment for deterioration reduction.
${RT}_{C1}$- Average total cost per unit time when the zero-inventory point *t*_1_occurs before or at the first trapezoidal break point *ω*_1_, (*t*_1_≤*ω*_1_).
${RT}_{C2}$- Average total cost per unit time when the zero-inventory point falls within the middle demand interval (*ω*_1_≤*t*_1_≤*ω*_2_).
${RT}_{C3}$- Average total cost per unit time when inventory depletes during the final declining demand phase (*ω*_2_≤*t*_1_≤*T*).

#### Assumptions

**Instantaneous replenishment:** Inventory replenishment is assumed to occur instantly, implying that stock is immediately available without any delay upon ordering.
**Amelioration rate:** The process of inventory amelioration follows a two-parameter Weibull distribution, mathematically represented as $E\left(t\right)=e{ft}^{f-1}$, where e is the scale parameter with a small positive value (0<e≪1) and f is the shape parameter (f>0); refer to (Hatibaruah and Saha^12^) and (Jayashri and Umamaheswari^13^).
**Deterioration rate:** Similarly, the deterioration of inventory is modeled using a two-parameter Weibull function given by $F\left(t\right)=c{dt}^{d-1}$, where c is a small positive scale parameter (0<c≪1), and d is the shape parameter (d>0).
**Preservation investment:** The retailer invests in preservation investment in order to curb deterioration. This investment is measured by the effectiveness of the reduction which is expressed as a reducing function *m*(*ξ*) = 1-*e*^-*qξ*^, in which the investment amount is written as *ξ* and *q* is (*q*> 0) is the sensitivity of the reduction rate of investment. This operation guarantees that 0 ≤ *m*(*ξ*) ≤ 1, which act in accordance with the physical limitations (refer to Dye and Hsieh^8^).
**Trapezoidal demand function:** The model assumes linear demand function, which changes both with time and price due to the effects of weibull-distributed amelioration and deterioration rates. This is the trapezoidal pattern of demand that occurs in three consecutive phases, the first period of rising demand, the second period which is characterized by a fixed demand, and the last period which is characterized with a falling demand. The demand is presented as a time dependent power law relationship of price that essentially describes the dynamics in every stage.

$$D\left(P,t\right)=\left\{ \begin{array}{c} \frac{a_{1}+b_{1}t}{P^{J}},0\leq t\leq \omega_{1}\\ \frac{a_{2}+b_{2}T}{P^{J}},\omega_{1}\leq t\leq \omega_{2}\\ \frac{a_{3}-b_{3}t}{P^{J}},\omega_{2}\leq t\leq T\; \end{array}\right.$$

Here, *a*_*i*_,*b*_*i*_ are constants for each time segment, *P* is the price, *J* is a price sensitivity parameter, and *T* is the cycle time (refer to Vandana and Srivastava^36^ and Xu et al.^37^).
**Cubic holding cost**: The holding cost is assumed to be a time-dependent function expressed as *αt*+*βt*^3^, where *α* > 0 and *β* > 0. The linear term *αt* reflects the regular storage expenses, while the cubic term *βt*^3^ accounts for the accelerated growth of costs due to deterioration, maintenance, or obsolescence over longer storage periods (refer to Thirugnanasambandam and Sivan^5^).
**Shortages and partial backlogging:** The model allows for stock shortages during the interval [*t*_1_,*T*], where only a portion of the unsatisfied demand is backordered. The proportion of demand that is backordered decreases with increased customer waiting time, reflecting time-sensitive customer behavior. To be more precise, a backordering rate is the exponential function *B*(*t*) = *e*^−*δ*(*T*-*t*)^, where *δ* > 0 is a backlogging parameter (*T*–*t*) is the waiting time between replenishment instances. This is based on the strategy suggested by Abad.^45^

### Mathematical formulation

In cases where inventory is prone to natural improvement and inevitable deterioration, the conventional models fail to capture the subtle changes throughout the time. The presented model is developed to represent the environment in which items do not just perish but also get better or even more valuable within a certain period of time. The replenishing of inventory occurs at the beginning of planning cycle [0,*T*], but after that it starts depleting with a slow process namely deterioration and amelioration. At the same time, a preservation investment system is also included, which functions as a control mechanism to reduce the speed of deterioration.

The consumer demand is modeled as a time and price-dependent trapezoidal, which is divided into three stages of development: a period of an increase to time *ω*_1_, the period of the constant rate to time *ω*_2_ and the period of the gradual decrease since then. The point in which the inventory becomes zero *t*_1_ indicates a critical point. Henceforth, backlogging of any unmet demand occurs partly, determined by the waiting time of customers willing to wait- This is mathematically expressed by a decay-based waiting function. The analysis into the demand-sensitive situations is a natural extension of the system by adding the demand variability and response to the backlog to the system dynamics.

To deal with this changing demand structure, the model is divided by the location of *t*_1_ falls in the demand intervals. The part of an inventory behavior in subintervals of [0,*T*] has a distinctive set of differential equations per case. These equations take into consideration the effect of intensity of preservation, nonlinear holding cost, and temporally weighted demand. This formulation unlike the static frameworks changes with time and is dynamically responsive to the internal system traits and external market shocks.

In order to make it clear, mathematical symbols, functions, and parameters employed in the system dynamics are given a clear definition, and then the case-wise differential equations are presented.

#### System dynamics

The rate of change in inventory level *I*(*t*) is described as a piecewise differential system:

(Equation 1) $$\frac{dI\left(t\right)}{dt}=\left\{ \begin{array}{cc} -\left(\left(1-m\left(\xi \right)\right)c{dt}^{d-1}-e{ft}^{f-1}\right)I\left(t\right)-D\left(P,t\right), & 0\leq t\leq t_{1}\\ -e^{-\delta \left(T-t\right)}D\left(P,t\right), & t_{1}\leq t\leq T \end{array}\right.$$

and the boundary condition: *I* (*t*_1_) = 0.

To be clear, the symbols used in the Equation 1 and the following case-wise formulations can be defined as follows. *I*(*t*) represents the quantity level of inventory at time *t*. The notations $c{dt}^{d-1}$ and $e{ft}^{f-1}$ denote the cumulative Weibull-controlled deterioration and amelioration rates. The *m*(*ξ*) is the preservation investment effect, which decreases the effective rate of deterioration. The trapezoidal demand is represented in the demand function *D* (*P*,*t*).The term *e*^−*δ*(*T*-*t*)^ is the partial backlogging behavior in the shortage interval [*t*1,*T*] where *δ* is the backlogging sensitivity parameter. These notations are consistent in Cases 1–3 and are applied in all cost expressions.

#### Case-based modeling approach

**Figure 2:**
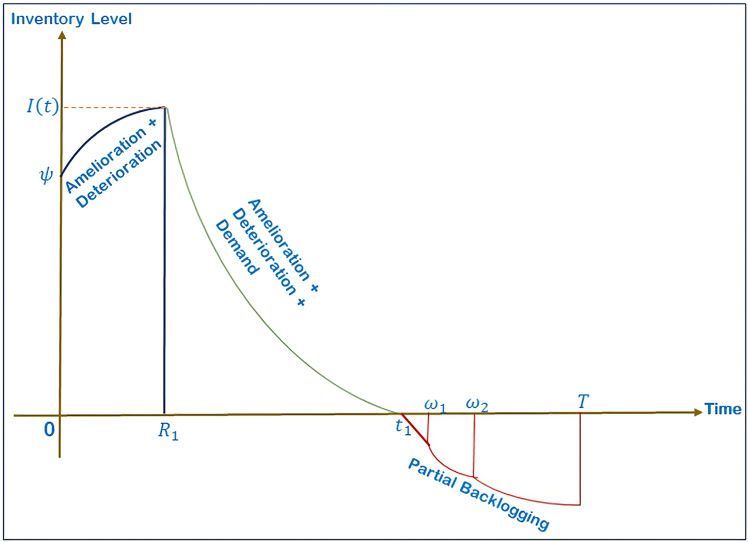
Case 1: 0 ≤ *t*_1_≤*ω*_1_

**Figure 3:**
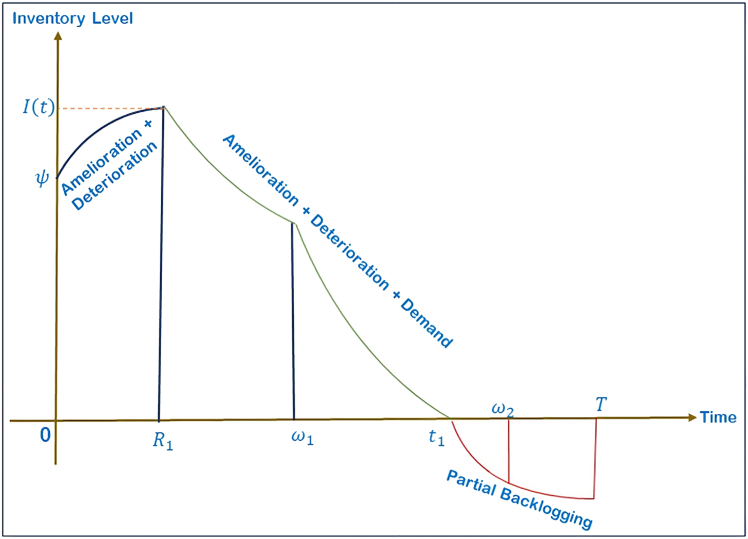
Case 2: *ω*_1_≤*t*_1_≤*ω*_2_

**Figure 4:**
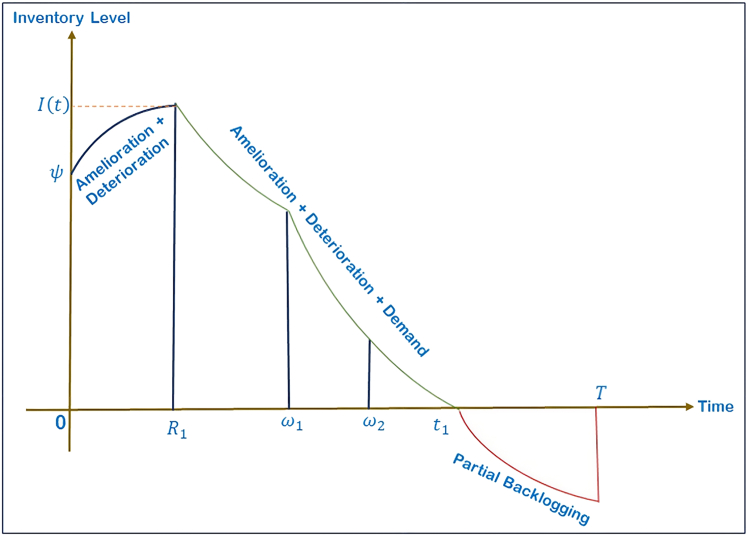
Case 3: *ω*_2_≤*t*_1_≤*T*

The model is designed to manage the seasonal fluctuations in demand and inventory behavior so that it will accommodate real-world demand fluctuations and inventory behavior, and three different cases are created as illustrated in Figures 2, 3, and 4, with each case representation represented under Case 1. 0≤*t*_1_≤*ω*_1_ shown in Figure 2, Case 2. *ω*_1_≤*t*_1_≤*ω*_2_ represented in Figure 3, and Case 3. *ω*_2_≤*t*_1_≤*T* illustrated in Figure 4.

Case 1 (0≤*t*_1_≤*ω*_1_): Since the inventory is evolving due to the presence of amelioration, deterioration and trapezoidal demand function that is time and price sensitive, controlled by preservation investment, the inventory will have a progressive decreasing trend over the period [0,*t*_1_], and finally takes an adiabatic approach at *t*_1_. Accordingly, Equation 1 yields:

(Equation 2) $$\begin{array}{cc} \frac{{dI}_{11}\left(t\right)}{dt}+\left(\left(1-m\left(\xi \right)\right)c{dt}^{d-1}-e{ft}^{f-1}\right)I_{11}\left(t\right)=0\text{,} & 0\leq t\leq R_{1} \end{array}$$

Equation 2 models the initial phase where inventory improves in quality due to amelioration and slightly decays due to deterioration. There is no active demand during this period.

(Equation 3) $$\begin{array}{cc} \frac{{dI}_{12}\left(t\right)}{dt}+\left(\left(1-m\left(\xi \right)\right)c{dt}^{d-1}-e{ft}^{f-1}\right)I_{12}\left(t\right)=-\left(\frac{a_{1}+b_{1}t}{P^{J}}\right)\text{,} & R_{1}\leq t\leq t_{1} \end{array}$$

Equation 3 governs the period of rising demand. The right-hand term $-\left(\frac{a_{1}+b_{1}t}{P^{J}}\right)$ is the time and price dependent rate of demand which decreases the inventory as consumer purchases.

(Equation 4) $$\begin{array}{cc} \frac{{dI}_{13}\left(t\right)}{dt}=-e^{-\delta \left(T-t\right)}\left(\frac{a_{1}+b_{1}t}{P^{J}}\right)\text{,} & t_{1}\leq t\leq \omega_{1} \end{array}$$

The shortage phase following the depletion of the inventory is represented by Equation 4 with a backlog demand at a declining rate, which is governed by the waiting-time factor *e*^−*δ*(*T*-*t*)^.

(Equation 5) $$\begin{array}{cc} \frac{{dI}_{14}\left(t\right)}{dt}=-e^{-\delta \left(T-t\right)}\left(\frac{a_{2}+b_{2}T}{P^{J}}\right)\text{,} & \omega_{1}\leq t\leq \omega_{2} \end{array}$$

Equation 5 models the constant demand period. Despite zero available inventory, customer demand continues at a steady rate, and only a portion of it is backlogged based on the waiting behavior.

(Equation 6) $$\begin{array}{cc} \frac{{dI}_{15}\left(t\right)}{dt}=-e^{-\delta \left(T-t\right)}\left(\frac{a_{3}-b_{3}t}{P^{J}}\right)\text{,} & \omega_{2}\leq t\leq T \end{array}$$

Equation 6 captures the declining demand phase where backorders and lost sales reduce gradually as the cycle approaches completion.

The boundary constraint at *t*_1_ = 0 enables the derivation of the following expressions for the inventory functions:

(Equation 7) $$\begin{array}{cc} I_{11}\left(t\right)=\psi \left(1+et^{f}-\left(1-m\left(\xi \right)\right)ct^{d}\right) & 0\leq t\leq R_{1} \end{array}$$

(Equation 8) $$\begin{array}{cc} I_{12}\left(t\right)=\frac{a_{1}}{P^{J}}\left(\frac{\left(m\left(\xi \right)-1\right)c\left(t_{1}^{d+1}-t^{d+1}\right)}{d+1}+\frac{e\left(t_{1}^{f+1}-t^{f+1}\right)}{f+1}+\left(t_{1}-t\right)\right)+\frac{b_{1}}{P^{J}}\left(\frac{\left(m\left(\xi \right)-1\right)c\left(t_{1}^{d+2}-t^{d+2}\right)}{d+2}+\frac{e\left(t_{1}^{f+2}-t^{f+2}\right)}{f+2}+\frac{\left({t_{1}}^{2}-t^{2}\right)}{2}\right) & R_{1}\leq t\leq t_{1} \end{array}$$

(Equation 9) $$\begin{array}{cc} I_{13}\left(t\right)=-\left(\frac{\left(\delta a_{1}+b_{1}\left(\delta \left(t-t_{1}\right)\right)-1\right)e^{\delta \left(\left(t-t_{1}\right)-T\right)}}{\delta^{2}P^{J}}\right) & t_{1}\leq t\leq \omega_{1} \end{array}$$

(Equation 10) $$\begin{array}{cc} I_{14}\left(t\right)=\frac{a_{2}+b_{2}T}{\delta P^{J}}\left(e^{\delta \left(\omega_{1}-T\right)}-e^{\delta \left(t-T\right)}\right)-\left(\frac{\left(\delta a_{1}+b_{1}\left(\delta \left(\omega_{1}-t_{1}\right)\right)-1\right)e^{\delta \left(\left(\omega_{1}-t_{1}\right)-T\right)}}{\delta^{2}P^{J}}\right) & \omega_{1}\leq t\leq \omega_{2} \end{array}$$

(Equation 11) $$\begin{array}{cc} I_{15}\left(t\right)=\left(\frac{\left(-\delta a_{3}+b_{3}\left(\delta \left(t-\omega_{2}\right)\right)-1\right)e^{\delta \left(\left(t-\omega_{2}\right)-T\right)}}{\delta^{2}P^{J}}\right)+\frac{a_{2}+b_{2}T}{\delta P^{J}}\left(e^{\delta \left(\omega_{1}-T\right)}-e^{\delta \left(\omega_{2}-T\right)}\right)-\left(\frac{\left(\delta a_{1}+b_{1}\left(\delta \left(\omega_{1}-t_{1}\right)\right)-1\right)e^{\delta \left(\left(\omega_{1}-t_{1}\right)-T\right)}}{\delta^{2}P^{J}}\right) & \omega_{2}\leq t\leq T \end{array}$$

#### Ordering cost = *CC*_*O*_

This is the fixed cost that would be incurred at any given time that an order is placed irrespective of the size of the order.

##### Ameliorating Cost

$${AA}_{c}=g_{1}\left(\int_{0}^{R_{1}}e{ft}^{f-1}I_{11}\left(t\right)dt+\int_{R_{1}}^{t_{1}}e{ft}^{f-1}I_{12}\left(t\right)dt\right)$$

See Equation A.1 in the appendix to get the detailed solution.

This expense reflects the reduction in inventory quality/value as time passes by as a result of favorable conditions.

##### Deteriorating cost

$${DD}_{c}=g_{2}\left(\int_{0}^{R_{1}}\left(1-m\left(\xi \right)\right)c{dt}^{d-1}I_{11}\left(t\right)dt+\int_{R_{1}}^{t_{1}}\left(1-m\left(\xi \right)\right)c{dt}^{d-1}I_{12}\left(t\right)dt\right)$$

The detailed solution may be seen in Equation A.2 in the Appendix.

This cost reflects on inventory loss in terms of value or quantity as it decays over time, after subtracting the preservation investment.

##### Holding cost

$${HH}_{c}=\int_{0}^{R_{1}}\left(\alpha t+\beta t^{3}\right)I_{11}\left(t\right)dt+\int_{R_{1}}^{t_{1}}\left(\alpha t+\beta t^{3}\right)I_{12}\left(t\right)dt$$

The detailed solution is available in Equation A.3 in appendix.

This cost includes the cost of holding and maintaining the inventory, which is increasing nonlinearly with time.

##### Backorder cost

$$T_{BB}=-g_{3}\int_{t_{1}}^{T}I\left(t\right)dt=-g_{3}\left(\int_{t_{1}}^{\omega_{1}}I_{13}\left(t\right)dt+\int_{\omega_{1}}^{\omega_{2}}I_{14}\left(t\right)dt+\int_{\omega_{2}}^{T}I_{15}\left(t\right)dt\right)$$

Refer to Equation A.4 in the appendix for the detailed solution.

The term measures the lost prices of backlogged demand at the shortage period where the customers are ready to wait in restocking.

##### Cost of lost sales

$$T_{LL}=g_{4}\left(\int_{t_{1}}^{\omega_{1}}\left(1-e^{\delta \left(t-T\right)}\right)\left(\frac{a_{1}+b_{1}t}{P^{J}}\right)dt+\int_{\omega_{1}}^{\omega_{2}}\left(1-e^{\delta \left(t-T\right)}\right)\left(\frac{a_{2}+b_{2}T}{P^{J}}\right)dt+\int_{\omega_{2}}^{T}\left(1-e^{\delta \left(t-T\right)}\right)\left(\frac{a_{3}-b_{3}t}{P^{J}}\right)dt\right)$$

Refer to Equation A.5 in the appendix for the detailed solution.

This shows the money loss on the sales that were not able to be fulfilled in the stock out phase.

##### Preservation investment cost

℘=ξT

This cost corresponds to the investment made to slow down deterioration and enhance product life through preservation investment.

##### Average total cost

$${RT}_{C1}=\frac{\left[{CC}_{O}+{AA}_{c}+{DD}_{c}+{HH}_{c}+T_{BB}+T_{LL}+℘\right]}{T}$$

This is the total average cost per unit time, integrating all relevant cost components over the cycle.

Case 2 (*ω*_1_≤*t*_1_≤*ω*_2_): inventory evolution under the influence of demand, amelioration, deterioration, and preservation is mathematically characterized by the following differential equations:

(Equation 12) $$\begin{array}{cc} \frac{{dI}_{21}\left(t\right)}{dt}+\left(\left(1-m\left(\xi \right)\right)c{dt}^{d-1}-e{ft}^{f-1}\right)I_{21}\left(t\right)=0\text{,} & 0\leq t\leq R_{1} \end{array}$$

Equation 12 models the initial phase where inventory improves in quality due to amelioration and slightly decays due to deterioration. No demand is active in this period.

(Equation 13) $$\begin{array}{cc} \frac{{dI}_{22}\left(t\right)}{dt}+\left(\left(1-m\left(\xi \right)\right)c{dt}^{d-1}-e{ft}^{f-1}\right)I_{22}\left(t\right)=-\left(\frac{a_{1}+b_{1}t}{P^{J}}\right)\text{,} & R_{1}\leq t\leq \omega_{1} \end{array}$$

Equation 13 governs the period of rising demand. The right-hand term $-\left(\frac{a_{1}+b_{1}t}{P^{J}}\right)$ represents the time and price-dependent demand rate, which reduces inventory proportionally to consumer purchases.

(Equation 14) $$\begin{array}{cc} \frac{{dI}_{23}\left(t\right)}{dt}+\left(\left(1-m\left(\xi \right)\right)c{dt}^{d-1}-e{ft}^{f-1}\right)I_{23}\left(t\right)=-\left(\frac{a_{2}+b_{2}T}{P^{J}}\right)\text{,} & \omega_{1}\leq t\leq t_{1} \end{array}$$

Equation 14 governs the period of constant demand. The right-hand term $-\left(\frac{a_{2}+b_{2}T}{P^{J}}\right)$ represents the time and price-dependent demand rate, which reduces inventory proportionally to consumer purchases.

(Equation 15) $$\begin{array}{cc} \frac{{dI}_{24}\left(t\right)}{dt}=-e^{-\delta \left(T-t\right)}\left(\frac{a_{2}+b_{2}T}{P^{J}}\right)\text{,} & t_{1}\leq t\leq \omega_{2} \end{array}$$

Equation 15 models the constant demand period. Despite zero available inventory, customer demand continues at a steady rate, and only a portion of it is backlogged based on the waiting behavior.

(Equation 16) $$\begin{array}{cc} \frac{{dI}_{25}\left(t\right)}{dt}=-e^{-\delta \left(T-t\right)}\left(\frac{a_{3}-b_{3}t}{P^{J}}\right)\text{,} & \omega_{2}\leq t\leq T \end{array}$$

Equation 16 captures the declining demand phase where backorders and lost sales reduce gradually as the cycle approaches completion.

The boundary constraint at *t*_1_ = 0 enables the derivation of the following expressions for the inventory functions:

(Equation 17) $$\begin{array}{cc} I_{21}\left(t\right)=\psi \left(1+et^{f}-\left(1-m\left(\xi \right)\right)ct^{d}\right) & 0\leq t\leq R_{1} \end{array}$$

(Equation 18) $$\begin{array}{cc} I_{22}\left(t\right)=\frac{a_{1}}{P^{J}}\left(\frac{\left(m\left(\xi \right)-1\right)c\left(\omega_{1}^{d+1}-t^{d+1}\right)}{d+1}+\frac{e\left(\omega_{1}^{f+1}-t^{f+1}\right)}{f+1}+\left(\omega_{1}-t\right)\right)+\frac{b_{1}}{P^{J}}\left(\frac{\left(m\left(\xi \right)-1\right)c\left(\omega_{1}^{d+2}-t^{d+2}\right)}{d+2}+\frac{e\left(\omega_{1}^{f+2}-t^{f+2}\right)}{f+2}+\frac{\left({\omega_{1}}^{2}-t^{2}\right)}{2}\right)+\frac{a_{2}+b_{2}T}{\delta P^{J}}\left(\frac{\left(m\left(\xi \right)-1\right)c\left(t_{1}^{d+1}-{\omega_{1}}^{d+1}\right)}{d+1}+\frac{e\left(t_{1}^{f+1}-{\omega_{1}}^{f+1}\right)}{f+1}+\left(t_{1}-\omega_{1}\right)\right) & R_{1}\leq t\leq \omega_{1} \end{array}$$

(Equation 19) $$\begin{array}{cc} I_{23}\left(t\right)=\frac{a_{2}+b_{2}T}{P^{J}}\left(\frac{\left(1-m\left(\xi \right)\right)c\left(t_{1}^{d+1}-t^{d+1}\right)}{d+1}+\frac{e\left(t_{1}^{f+1}-t^{f+1}\right)}{f+1}+\left(t_{1}-t\right)\right) & \omega_{1}\leq t\leq t_{1} \end{array}$$

(Equation 20) $$\begin{array}{cc} I_{24}\left(t\right)=\frac{a_{2}+b_{2}T}{\delta P^{J}}\left(e^{\delta \left(t_{1}-T\right)}-e^{\delta \left(t-T\right)}\right) & t_{1}\leq t\leq \omega_{2} \end{array}$$

(Equation 21) $$\begin{array}{cc} I_{25}\left(t\right)=\left(\frac{\left(-\delta a_{3}+b_{3}\left(\delta \left(t-\omega_{2}\right)\right)-1\right)e^{\delta \left(\left(t-\omega_{2}\right)-T\right)}}{\delta^{2}P^{J}}\right)+\frac{a_{2}+b_{2}T}{\delta P^{J}}\left(e^{\delta \left(t_{1}-T\right)}-e^{\delta \left(\omega_{2}-T\right)}\right) & \omega_{2}\leq t\leq T \end{array}$$

#### Ordering cost = *CC*_*O*_

##### Ameliorating Cost

$${AA}_{c}=g_{1}\left(\int_{0}^{R_{1}}e{ft}^{f-1}I_{21}\left(t\right)dt+\int_{R_{1}}^{\omega_{1}}e{ft}^{f-1}I_{22}\left(t\right)dt+\int_{\omega_{1}}^{t_{1}}e{ft}^{f-1}I_{23}\left(t\right)dt\right)$$

Refer to Equation A.6 in the Appendix for the detailed solution.

##### Deteriorating cost

$${DD}_{c}=g_{2}\left(\int_{0}^{R_{1}}\left(1-m\left(\xi \right)\right)c{dt}^{d-1}I_{21}\left(t\right)dt+\int_{R_{1}}^{\omega_{1}}\left(1-m\left(\xi \right)\right)c{dt}^{d-1}I_{22}\left(t\right)dt+\int_{\omega_{1}}^{t_{1}}\left(1-m\left(\xi \right)\right)c{dt}^{d-1}I_{23}\left(t\right)dt\right)$$

Refer to Equation A.7 in the Appendix for the detailed solution.

##### Holding cost

$${HH}_{c}=\int_{0}^{R_{1}}\left(\alpha t+\beta t^{3}\right)I_{21}\left(t\right)dt+\int_{R_{1}}^{\omega_{1}}\left(\alpha t+\beta t^{3}\right)I_{22}\left(t\right)dt+\int_{\omega_{1}}^{t_{1}}\left(\alpha t+\beta t^{3}\right)I_{23}\left(t\right)dt$$

Refer to Equation A.8 in the Appendix for the detailed solution.

##### Backorder cost

$$T_{BB}=-g_{3}\int_{t_{1}}^{T}I\left(t\right)dt=-g_{3}\left(\int_{t_{1}}^{\omega_{2}}I_{24}\left(t\right)dt+\int_{\omega_{2}}^{T}I_{25}\left(t\right)dt\right)$$

Refer to Equation A.9 in the Appendix for the detailed solution.

##### Cost of lost sales

$$T_{LL}=g_{4}\left(\int_{t_{1}}^{\omega_{2}}\left(1-e^{\delta \left(t-T\right)}\right)\left(\frac{a_{2}+b_{2}T}{P^{J}}\right)dt+\int_{\omega_{2}}^{T}\left(1-e^{\delta \left(t-T\right)}\right)\left(\frac{a_{3}-b_{3}t}{P^{J}}\right)dt\right)$$

Refer to Equation A.10 in the Appendix for the detailed solution.

##### Preservation investment cost

℘=ξT

##### Average total cost

$${RT}_{C2}=\frac{\left[{CC}_{O}+{AA}_{c}+{DD}_{c}+{HH}_{c}+T_{BB}+T_{LL}+℘\right]}{T}$$

Case 3 (*ω*_2_≤*t*_1_≤*T*): The mathematical representation of inventory variation over time, incorporating demand, preservation, amelioration and degradation effects, is outlined through the following differential equations:

(Equation 22) $$\begin{array}{cc} \frac{{dI}_{31}\left(t\right)}{dt}+\left(\left(1-m\left(\xi \right)\right)c{dt}^{d-1}-e{ft}^{f-1}\right)I_{31}\left(t\right)=0\text{,} & 0\leq t\leq R_{1} \end{array}$$

Equation 22 models the initial phase where inventory improves in quality due to amelioration and slightly decays due to deterioration. No demand is active in this period.

(Equation 23) $$\begin{array}{cc} \frac{{dI}_{32}\left(t\right)}{dt}+\left(\left(1-m\left(\xi \right)\right)c{dt}^{d-1}-e{ft}^{f-1}\right)I_{32}\left(t\right)=-\left(\frac{a_{1}+b_{1}t}{P^{J}}\right)\text{,} & R_{1}\leq t\leq \omega_{1} \end{array}$$

Equation 23 governs the period of rising demand. The right-hand term $-\left(\frac{a_{1}+b_{1}t}{P^{J}}\right)$ represents the time and price-dependent demand rate, which reduces inventory proportionally to consumer purchases.

(Equation 24) $$\begin{array}{cc} \frac{{dI}_{33}\left(t\right)}{dt}+\left(\left(1-m\left(\xi \right)\right)c{dt}^{d-1}-e{ft}^{f-1}\right)I_{33}\left(t\right)=-\left(\frac{a_{2}+b_{2}T}{P^{J}}\right)\text{,} & \omega_{1}\leq t\leq \omega_{2} \end{array}$$

Equation 24 governs the period of constant demand. The right-hand term $-\left(\frac{a_{2}+b_{2}T}{P^{J}}\right)$ represents the time and price-dependent demand rate, which reduces inventory proportionally to consumer purchases.

(Equation 25) $$\begin{array}{cc} \frac{{dI}_{34}\left(t\right)}{dt}+\left(\left(1-m\left(\xi \right)\right)c{dt}^{d-1}-e{ft}^{f-1}\right)I_{34}\left(t\right)=-\left(\frac{a_{3}-b_{3}t}{P^{J}}\right)\text{,} & \omega_{2}\leq t\leq t_{1} \end{array}$$

Equation 25 governs the period of declining demand. The right-hand term $-\left(\frac{a_{3}-b_{3}t}{P^{J}}\right)$ represents the time and price-dependent demand rate, which reduces inventory proportionally to consumer purchases.

(Equation 26) $$\begin{array}{cc} \frac{{dI}_{35}\left(t\right)}{dt}=-e^{-\delta \left(T-t\right)}\left(\frac{a_{3}-b_{3}t}{P^{J}}\right)\text{,} & t_{1}\leq t\leq T \end{array}$$

Equation 26 captures the declining demand phase where backorders and lost sales reduce gradually as the cycle approaches completion.

The boundary constraint at *t*_1_ = 0 enables the derivation of the following expressions for the inventory functions:

(Equation 27) $$\begin{array}{cc} I_{31}\left(t\right)=\psi \left(1+et^{f}-\left(1-m\left(\xi \right)\right)ct^{d}\right) & 0\leq t\leq R_{1} \end{array}$$

(Equation 28) $$I_{32}\left(t\right)=\frac{a_{1}}{P^{J}}\left(\frac{\left(m\left(\xi \right)-1\right)c\left(\omega_{1}^{d+1}-t^{d+1}\right)}{d+1}+\frac{e\left(\omega_{1}^{f+1}-t^{f+1}\right)}{f+1}+\left(\omega_{1}-t\right)\right)+\frac{b_{1}}{P^{J}}\left(\frac{\left(m\left(\xi \right)-1\right)c\left(\omega_{1}^{d+2}-t^{d+2}\right)}{d+2}+\frac{e\left(\omega_{1}^{f+2}-t^{f+2}\right)}{f+2}+\frac{\left({\omega_{1}}^{2}-t^{2}\right)}{2}\right)+\frac{a_{2}+b_{2}T}{\delta P^{J}}\left(\frac{\left(m\left(\xi \right)-1\right)c\left(\omega_{2}^{d+1}-{\omega_{1}}^{d+1}\right)}{d+1}+\frac{e\left(\omega_{2}^{f+1}-{\omega_{1}}^{f+1}\right)}{f+1}+\left(\omega_{2}-\omega_{1}\right)\right)+\frac{a_{3}}{P^{J}}\left(\frac{\left(m\left(\xi \right)-1\right)c\left(t_{1}^{d+1}-{\omega_{2}}^{d+1}\right)}{d+1}+\frac{e\left(t_{1}^{f+1}-{\omega_{2}}^{f+1}\right)}{f+1}+\left(t_{1}-\omega_{2}\right)\right)+\frac{b_{3}}{P^{J}}\left(\frac{\left(m\left(\xi \right)-1\right)c\left(t_{1}^{d+2}-{\omega_{2}}^{d+2}\right)}{d+2}-\frac{e\left(t_{1}^{f+2}-{\omega_{2}}^{f+2}\right)}{f+2}-\frac{\left({t_{1}}^{2}-{\omega_{2}}^{2}\right)}{2}\right)\;R_{1}\leq t\leq \omega_{1}$$

(Equation 29) $$\begin{array}{cc} I_{33}\left(t\right)=\frac{a_{2}+b_{2}T}{P^{J}}\left(\frac{\left(1-m\left(\xi \right)\right)c\left(\omega_{2}^{d+1}-t^{d+1}\right)}{d+1}+\frac{e\left(\omega_{2}^{f+1}-t^{f+1}\right)}{f+1}+\left(\omega_{2}-t\right)\right)+\frac{a_{3}}{P^{J}}\left(\frac{\left(m\left(\xi \right)-1\right)c\left(t_{1}^{d+1}-{\omega_{2}}^{d+1}\right)}{d+1}+\frac{e\left(t_{1}^{f+1}-{\omega_{2}}^{f+1}\right)}{f+1}+\left(t_{1}-\omega_{2}\right)\right)+\frac{b_{3}}{P^{J}}\left(\frac{\left(1-m\left(\xi \right)\right)c\left(t_{1}^{d+2}-{\omega_{2}}^{d+2}\right)}{d+2}-\frac{e\left(t_{1}^{f+2}-{\omega_{2}}^{f+2}\right)}{f+2}-\frac{\left({t_{1}}^{2}-{\omega_{2}}^{2}\right)}{2}\right) & \omega_{1}\leq t\leq \omega_{2} \end{array}$$

(Equation 30) $$\begin{array}{cc} I_{34}\left(t\right)=\frac{a_{3}}{P^{J}}\left(\frac{\left(1-m\left(\xi \right)\right)c\left(t_{1}^{d+1}-t^{d+1}\right)}{d+1}+\frac{e\left(t_{1}^{f+1}-t^{f+1}\right)}{f+1}+\left(t_{1}-t\right)\right)+\frac{b_{3}}{P^{J}}\left(\frac{\left(1-m\left(\xi \right)\right)c\left(t_{1}^{d+2}-t^{d+2}\right)}{d+2}-\frac{e\left(t_{1}^{f+2}-t^{f+2}\right)}{f+2}-\frac{\left({t_{1}}^{2}-t^{2}\right)}{2}\right) & \omega_{2}\leq t\leq t_{1} \end{array}$$

(Equation 31) $$\begin{array}{cc} I_{35}\left(t\right)=\left(\frac{\left(-\delta a_{3}+b_{3}\left(\delta \left(t-t_{1}\right)\right)-1\right)e^{\delta \left(\left(t-t_{1}\right)-T\right)}}{\delta^{2}P^{J}}\right) & t_{1}\leq t\leq T \end{array}$$

#### Ordering cost = *CC*_*O*_

##### Ameliorating Cost

$${AA}_{c}=g_{1}\left(\int_{0}^{R_{1}}e{ft}^{f-1}I_{31}\left(t\right)dt+\int_{R_{1}}^{\omega_{1}}e{ft}^{f-1}I_{32}\left(t\right)dt+\int_{\omega_{1}}^{\omega_{2}}e{ft}^{f-1}I_{33}\left(t\right)dt+\int_{\omega_{2}}^{t_{1}}e{ft}^{f-1}I_{34}\left(t\right)dt\right)$$

The detailed solution is found in Equation A.11 in the appendix.

##### Deteriorating cost

$${DD}_{c}=g_{2}\left(\int_{0}^{R_{1}}\left(1-m\left(\xi \right)\right)c{dt}^{d-1}I_{31}\left(t\right)dt+\int_{R_{1}}^{\omega_{1}}\left(1-m\left(\xi \right)\right)c{dt}^{d-1}I_{32}\left(t\right)dt+\int_{\omega_{1}}^{\omega_{2}}\left(1-m\left(\xi \right)\right)c{dt}^{d-1}I_{33}\left(t\right)dt+\int_{\omega_{2}}^{t_{1}}\left(1-m\left(\xi \right)\right)c{dt}^{d-1}I_{34}\left(t\right)dt\right)$$

Refer to Equation A.12 in the appendix for the detailed solution.

##### Holding cost

$${HH}_{c}=\int_{0}^{R_{1}}\left(\alpha t+\beta t^{3}\right)I_{31}\left(t\right)dt+\int_{R_{1}}^{\omega_{1}}\left(\alpha t+\beta t^{3}\right)I_{32}\left(t\right)dt+\int_{\omega_{1}}^{\omega_{2}}\left(\alpha t+\beta t^{3}\right)I_{33}\left(t\right)dt+\int_{\omega_{2}}^{t_{1}}\left(\alpha t+\beta t^{3}\right)I_{34}\left(t\right)dt$$

Refer to Equation A.13 in the appendix for the detailed solution.

##### Backorder cost

$$T_{BB}=-g_{3}\int_{t_{1}}^{T}I\left(t\right)dt=-g_{3}\left(\int_{t_{1}}^{T}I_{25}\left(t\right)dt\right)$$

Refer to Equation A.14 in the appendix for the detailed solution.

##### Cost of lost sales

$$T_{LL}=g_{4}\left(\int_{t_{1}}^{T}\left(1-e^{\delta \left(t-T\right)}\right)\left(\frac{a_{3}-b_{3}t}{P^{J}}\right)dt\right)$$

Refer to Equation A.15 in the appendix for the detailed solution.

##### Preservation investment cost

℘=ξT

##### Average total cost

$${RT}_{C3}=\frac{\left[{CC}_{O}+{AA}_{c}+{DD}_{c}+{HH}_{c}+T_{BB}+T_{LL}+℘\right]}{T}$$

### Optimization methodology

The proposed inventory model that combines seasonal variability and simultaneous product improvement and degradation with nonlinear components of costs needs a flexible and adaptive strategy to solve. Multidimensional and nonlinear formulations of this nature are hard to solve by conventional optimization techniques. To address this, the proposed research will use two biologically inspired algorithms to simulate natural behaviors to explore and exploit the solution space in depth. The GA and the KHA represent these two and they provide different strengths in navigating in complex optimization landscapes.

#### Genetic algorithm for inventory decision optimization

GA proposed by Holland^43^ is inspired by biological evolution, where populations evolve over a period of time by mechanisms like reproduction and mutation. In this method the inventory policies determined by the decision variables such as cycle time, preservation investment and pricing are presented as solution strings or “chromosome.”

**Figure 5:**
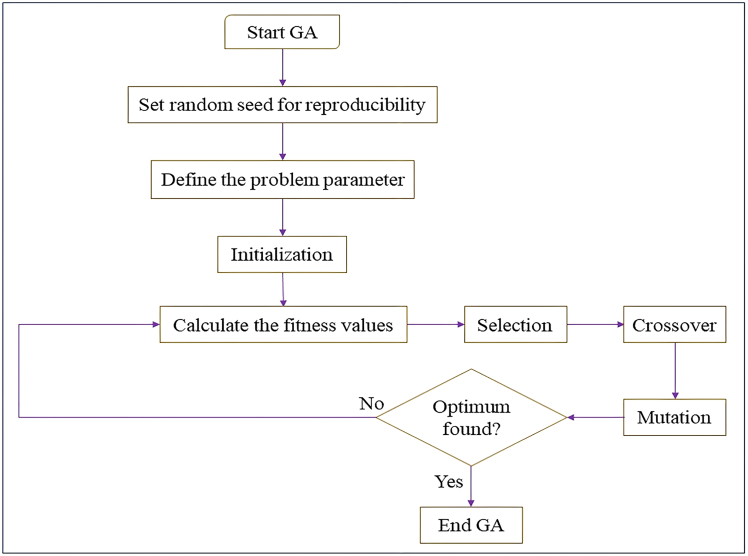
Sequential optimization framework using genetic algorithm

The algorithm is started by a starting population of randomly generated policies. Repeatedly new generations are formed by means of three fundamental operations:

• **Selection**, which favors cost-effective solutions on the basis of the fitness (average total cost minimization).
• **Crossover**, also known as hybrid-combination, involves the use of traits of two parent solutions to generate new ones.
• **Mutation**, the presence of small modifications to seek new opportunities and to prevent the rapid convergence.
Penalty terms are used to handle feasibility constraints, which deter the violations of negative investment or impracticable backlogs. The adaptive learning behavior of GA allows it to determine the best policies in the presence of strong policies even without accurate knowledge of the environment namely in Figure 5.

### Krill herd metaheuristic for inventory optimization

In order to verify and support the solutions that have been achieved with the help of GA, the KHA, suggested by Gandomi and Alavi,^44^ is also used. The idea behind this technique is based on the movement of krill swarms whereby the interactions with neighbors determine the direction of each individual, the search of food, and random movement.

The solutions in the KHA framework become krill’s and their placement in the solution space is modified with the aid of three behavioral components:

• **Neighbor influence**, reflecting local competition and cooperation.
• **Foraging action**, driven by memory and global performance trends.
• **Random diffusion**, to introduce diversity and broaden search reach.

**Figure 6:**
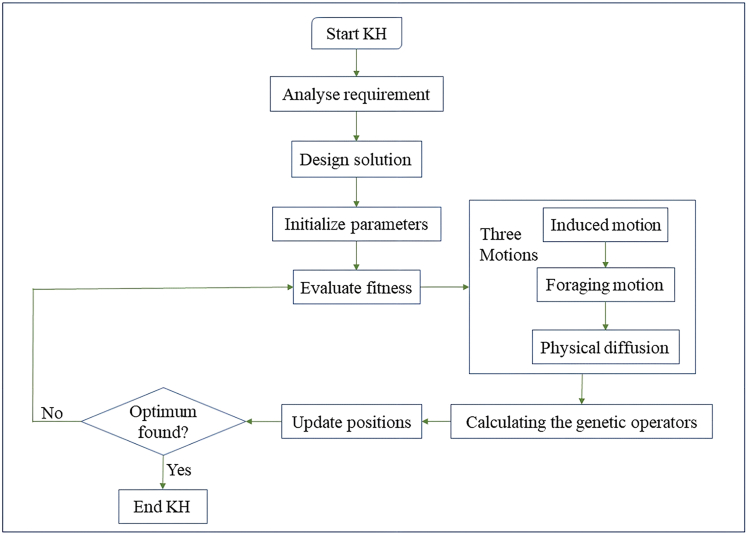
Sequential optimization framework using krill herd algorithm

As captured in Figure 6, KHA dynamically updates each solution position based on its performance in minimizing total inventory cost. Its compromise of guided search and random motion prevents it to get stuck in local optima, and this is essential in the inventory systems where there is a complex trade-off between deterioration, preservation, and replenishment.

#### Computational procedure

The performance of the GA and KHA algorithms is independent and not hybridized in MATLAB to assess the performance of the algorithms separately. The parameters like the number of people, iteration, and learning coefficients are adjusted by trial experiments. Their outputs are contrasted with respect to convergence behavior, robustness of solutions and time of computation of various demand patterns (rising, stable, and falling).

This dual pronged solution approach makes sure that the model enjoys both the evolutionary learning and swarm intelligence so that effective and dependable inventory policy is achieved that is applicable in dynamic and perishable settings.

### Numerical examples

**Figure 7:**
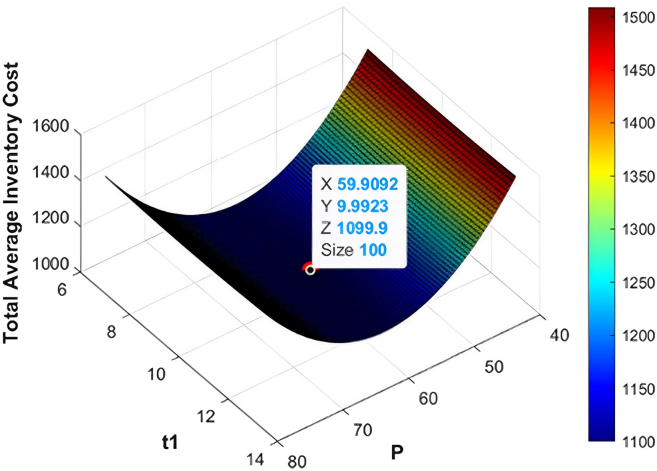
Optimal solutions to the scenario of example 1 with shortage are visualized

**Figure 8:**
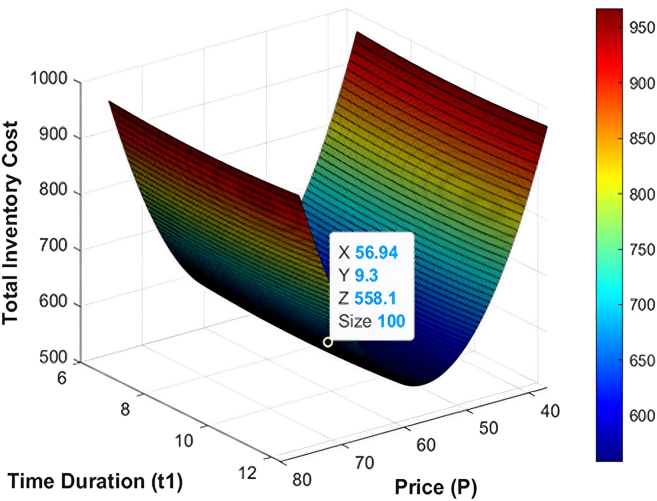
Optimal solutions to the scenario of example 1 without shortage are visualized

**Figure 9:**
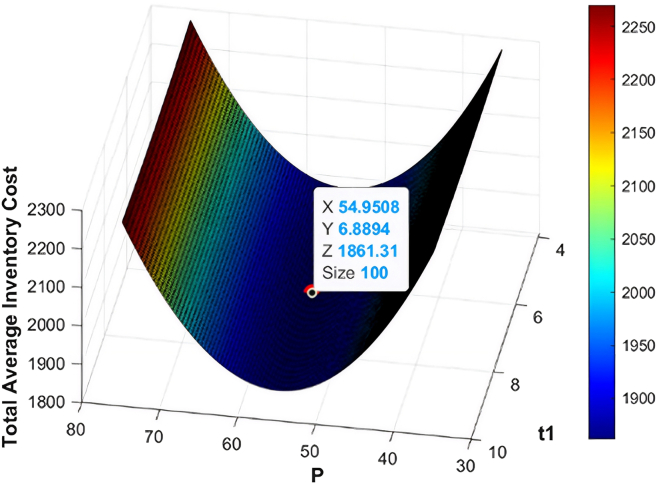
Optimal solutions to the scenario of example 2 with shortage are visualized

**Figure 10:**
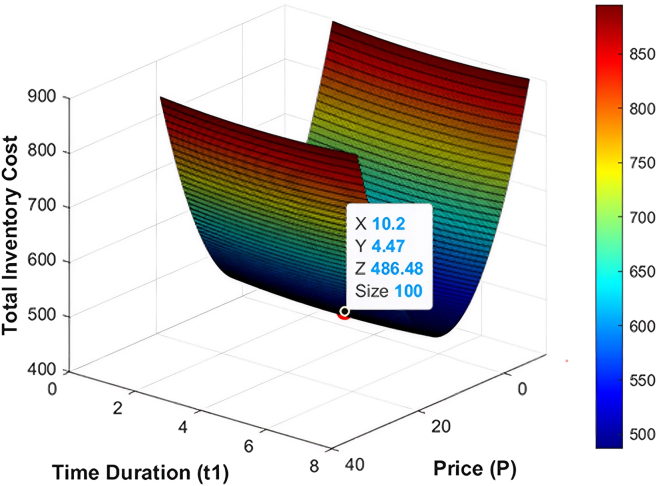
Optimal solutions to the scenario of example 2 without shortage are visualized

**Figure 11:**
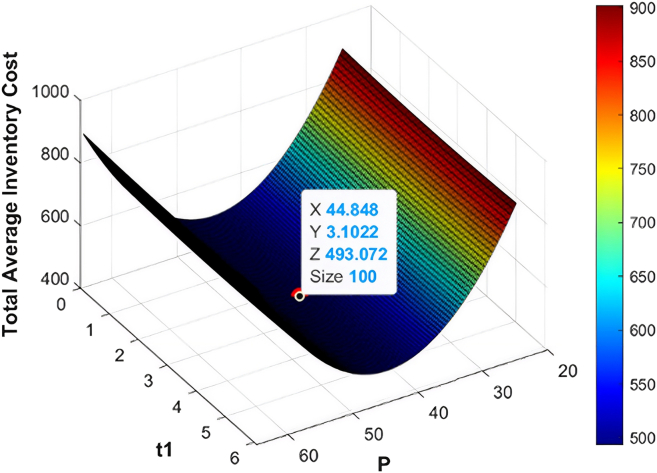
Optimal solutions to the scenario of example 3 with shortage are visualized

**Figure 12:**
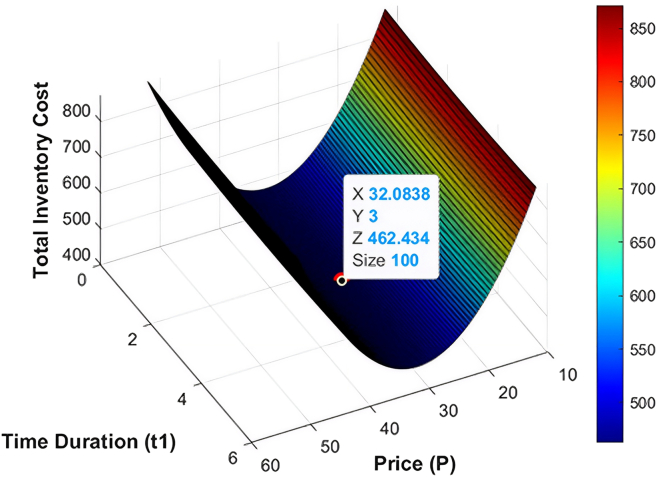
Optimal solutions to the scenario of example 3 without shortage are visualized

The numerical data employed in this research have been based on the existing literature on the deteriorating and ameliorating inventory models to have realistic parameterization and comparability. The same findings of GA and KHA suggest the convex and unimodal character of avaerage total cost, which proves that it converts to a global optimum. The data are not industry-specific but literature based, borrowed off previous studies that deal with similar deteriorating and ameliorating models of inventory. Figures 7, 8, 9, 10, 11, and 12 therefore record the optimum selling price, *x* = *P*, optimal cycle time, *y* = *t*_1_, and the overall composite cost ${z=RT}_{C1}$, which is in continuation with the previous discussion.

#### Example 1

To demonstrate the relevance of the suggested inventory framework, the in-depth numerical evaluation was performed using the GA and KHA optimization methods. All the parameters are stated in consistent units that are based on time, cost in months, cost in US dollars ($), and demand in units to have physical and dimensional consistency. The values of the parameters applied in this section are modified to the available literature to depict the real-world livestock inventory conditions. The parameters of inventory system were as follows: ordering cost was set as *CC*_*O*_ = 200$ per cycle. Amelioration effect of inventory items was modeled with two parameter Weibull distribution with e = 0.3 and f = 0.6, the effectiveness of preservation investment was determined by a sensitivity coefficient *q* = 1.2. Concurrently, the degradation had a Weibull pattern characterized by the c = 0.04 and d = 2.

The demand function was based on a trapezoidal structure, with the following three stages, increasing demand with coefficients *a*_1_ = 100 units and *b*_1_ = 5 units per month, constant demand with *a*_2∗_ = *a*_2_+*b*_2_*T* = 120 units, and decreasing demand with coefficients *a*_3_ = 220 units and *b*_3_ = 10 units per month. The elasticity of demand with respect to price was regulated by parameter *J* = 1.5. The holding cost of inventory was represented by a cubic cost equation, where *α* = 2$ is the coefficient of *β* = 0.0003$ in units of a cycle cubed. Backlogging mechanism, which was a partial demand backordering in times of shortages, was added with *δ* = 0.03. In addition, the unit amelioration cost (*g*_1_), deterioration cost (*g*_2_), shortage cost (*g*_3_), and the penalty cost of lost sales (*g*_4_) were assigned to 20$, 3$, 10$, and 8$ per unit respectively. The cycle time was set and the time segmentations were *ω*_1_ = 6 months and *ω*_2_ = 10 months, and the initial non-demand time was *R*_1_ = 1.2 months and the overall time of the cycle was *T* = 12 months.

When running the optimization processes, the GA and the KHA algorithms always found the optimal replenishment strategy. To be more precise, the optimal quantity of replenishment under the shortage condition was established at *ψ* = 551.68 units per cycle, the preservation investment was determined at *ξ* = 131.41$ per cycle. The best selling price was calculated to be *P* = 59.91$ per unit and inventory deterioration is calculated to take place at *t*_1_ = 9.99 and give a total composite cost of ${RT}_{C1}$ = 1099.89$ with minimum cost. In the no-shortage scenario, the optimal level of order was reduced to*ψ* = 539.75 units per cycle, preservation investment of *ξ* = 126.37$ per cycle and optimal selling price of *P* = 56.94$ per unit with depletion of inventory at *t*_1_ = 9.3 months, which incurred a lower total composite cost of ${RT}_{C1}$ = 558.1$ per cycle. The results of both algorithms in terms of optimization are posted in Table 2. Also, the connections between replenishment time, selling price and average total cost are displayed in Figures 7 and 8 which provide obvious operational understanding of the interactions of the inventory system in the most favorable circumstances.

**Table 2:** Optimized inventory metrics using GA and KHA

S. no.	Shortage	Optimization method	Order quantity ***ψ*** (units/cycle)	Preservation investment ***ξ*** ($/cycle)	Selling price *P* ($/units)	Replenishment time ***t***_1_ (months)	Average total cost ${RT}_{C1}$ ($/cycle)
1	Yes	GA	551.68	131.41	59.91	9.99	1,099.89
2		KHA	551.68	131.41	59.91	9.99	1,099.89
3	No	GA	539.75	126.37	56.94	9.3	558.1
4		KHA	539.75	126.37	56.94	9.3	558.1

#### Example 2

Another practical case was evaluated to further evaluate the performance of the proposed model under changed conditions of demand. The parameters *a*_3_ = 160 units and *b*_3_ = 5 units per month were used to redefine the declining demand phase in this case. The breakpoints of the trapezoidal demand pattern were determined to be *ω*_1_ = 2 months and *ω*_2_ = 8 months, which indicated a previous adjustment of a trapezoidal demand phase than in the former example. Other system parameters remained the same as in example 1.

After optimization of the model with both GA and KHA methods, there was a convergence in solution of the model. The optimal replenishment level was calculated to be *ψ* = 546.63 units per cycle, and a preservation investment of *ξ* = 120.35$ which was actually able to reduce deterioration during the cycle. The best selling price was determined as *P* = 54.95$ p and the inventory depleted at *t*_1_ = 6.89 months and the minimal overall composite cost was ${RT}_{C2}$ = 1861.31$ per cycle. In the without-shortage case, the optimal order quantity decreased to *ψ* = 450.3 units per cycle, with a preservation investment of *ξ* = 110.1$ per cycle and optimal selling price of *P* = 10.2$ per unit, while inventory depletion occurred at *t*_1_ = 4.47 months. These optimized parameters resulted in a minimum total composite cost of ${RT}_{C2}$ = 486.488$ per cycle which proves that the model is effective in optimization of cost-effective inventory management. Table 3 shows the optimum solution measures of the two algorithms. Moreover, Figures 9 and 10 offer the graphical representation of performance of the inventory system throughout the cycle, data of which demonstrates the impact of the adjusted demand structure in the relationship between the timing of the replenishment, prices and average total cost behavior.

**Table 3:** Optimal results for example 2 using GA and KHA

S. No.	Shortage	Optimization method	Order quantity ***ψ*** (units/cycle)	Preservation investment ***ξ*** ($/cycle)	Selling price *P* ($/units)	Replenishment time ***t***_1_ (months)	Average total cost ${RT}_{C2}$ ($/cycle)
1	Yes	GA	546.63	120.35	54.95	6.89	1,861.31
2		KHA	546.63	120.35	54.95	6.89	1,861.31
3	No	GA	450.3	110.1	10.2	4.47	486.48
4		KHA	450.3	110.1	10.2	4.47	486.48

#### Example 3

The third case was used to study how the model will respond when the model loses inventory earlier in the falling demand curve. In the present example, the parameters of the final demand stage were changed to *a*_3_ = 108 units and *b*_3_ = 1 unit per month representing a slower drop in demand. The trapezoidal demand structure was redefined where the phase breakpoints were defined as *ω*_1_ = 2 months and *ω*_2_ = 3 months which form a much shorter constant demand phase than was previously found.

Both the GA and KHA methods brought the same results, which prove the stability and validity of the offered method. In the case of shortage, the optimum amount of order was *ψ* = 469.56 units per cycle with the preservation investment of *ξ* = 105.25$ per cycle. The optimum selling price was *P* = 44.84$ per unit, and the depletion of inventory was relatively early at *t*_1_ = 3.10 months, which means that the minimum total composite cost is ${RT}_{C3}$ = 493.07$ per cycle. The optimal order quantity decreased to *ψ* = 440.12 units per cycle, preservation investment is *ξ* = 105.11$ per cycle and optimal selling price is *P* = 32.08$ per unit, and a reduction in inventory depletion was at *t*_1_ = 3.0 months resulting in a lower total composite cost ${RT}_{C3}$ = 462.43$ per cycle. A Table 4 shows a summary of the optimized results of the example 3, which validates the results of both optimization methods. In addition, Figures 11 and 12 depict the working behavior of the system showing the effect of reduced demand period on the decision of replenishment and the overall cost performance. The numerical illustration is based on the data that are not of a particular case but rather on the literature on parameter values of the typical livestock operations. This model can be confirmed by future work based on real industrial data.

**Table 4:** Optimal solution metrics for example 3 using GA and KHA

S. no.	Shortage	Optimization method	Order quantity ***ψ*** (units/cycle)	Preservation investment ***ξ*** ($/cycle)	Selling price *P* ($/units)	Replenishment time ***t***_1_ (months)	Average total cost ${RT}_{C3}$ ($/cycle)
1	Yes	GA	469.56	105.25	44.84	3.10	493.07
2		KHA	469.56	105.25	44.84	3.10	493.07
3	No	GA	440.12	105.11	32.08	3.0	462.43
4		KHA	440.12	105.11	32.08	3.0	462.43

To further enhance the comparative analysis, baseline performance is provided in both with-shortage and without-shortage scenarios (see Tables 2, 3, and 4; Figures 7, 8, 9, 10, 11, and 12). This comparison shows the real utility of having preservation investment and partial backlogging as part of the proposed livestock inventory.

### Sensitivity analysis

**Figure 13:**
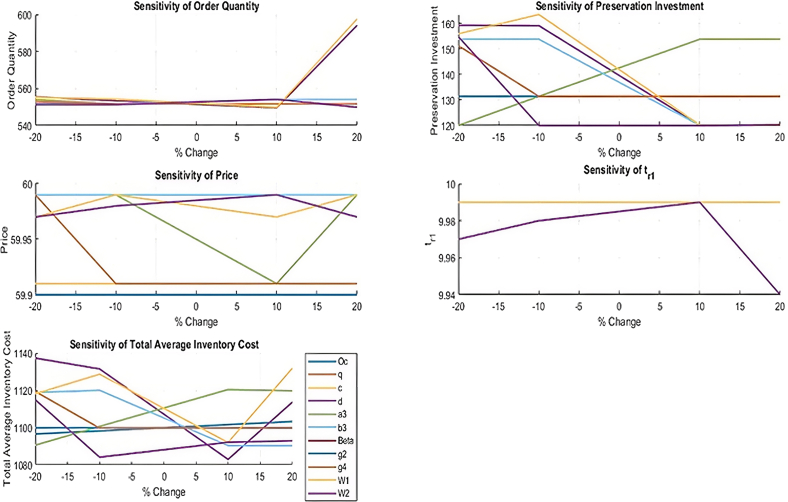
Effects of change in parameters on order quantity, preservation investment, price, replenishment time as well as average total inventory cost

Example 1 was conducted to a detailed sensitivity analysis to examine the influence of the changes in the critical parameters on the total average cost of inventory and the key variables in decision making. The parameters were individually modified by −20%, −10%, +10%, and +20% when the rest of the parameters were kept constant to test the properties of individual parameters. The resulting changes in order quantity (*ψ*), preservation investment (*ξ*), selling price (*P*), replenishment time (*t*_1_), and total average inventory cost $\left({RT}_{C1}\right)$ are articulately tabulated in Table 5. Moreover, the graph patterns related to such changes are provided in Figure 13 in which the model is graphically interpreted showing the reaction on the changes in operation conditions. Table 5 results reveal cost sensitive and stable parameters that give greater insights into the effect of the managerial choices on the overall cost efficiency of the livestock inventory operations.

**Table 5:** Sensitivity analysis summary of how the decision variables and average total cost vary as a result of alterations in the parameters in terms of example 1

Parameter name	Initial setting	Adjustment range (%)	*ψ*	***ξ***	*P*	*t*_1_	Average total cost (***ṪI***_***c***_)
*CC*_*O*_	200	−20%	551.68	131.408	59.91	9.99	1,096.56
		−10%	551.68	131.408	59.91	9.99	1,098.2
		+10%	551.68	131.408	59.91	9.99	1,101.56
		+20%	551.68	131.408	59.91	9.99	1,103.23
*q*	1.2	−20%	551.68	131.41	59.91	9.99	1,099.89
		−10%	551.68	131.41	59.91	9.99	1,099.89
		+10%	551.68	131.41	59.91	9.99	1,099.89
		+20%	551.68	131.41	59.91	9.99	1,099.89
c	0.04	−20%	551.68	131.41	59.91	9.99	1,099.89
		−10%	551.68	131.41	59.91	9.99	1,099.89
		+10%	551.68	131.41	59.91	9.99	1,099.89
		+20%	551.68	131.41	59.91	9.99	1,099.89
d	2	−20%	555.55	159.18	59.97	9.99	1,137.23
		−10%	553.33	159.05	59.99	9.99	1,131.42
		+10%	549.62	120.06	59.99	9.99	1,082.94
		+20%	594.11	120.03	59.99	9.99	1,113.59
*a*_3_	220	−20%	554.05	120.02	59.99	9.99	1,090.59
		−10%	551.68	131.41	59.99	9.99	1,100.6
		+10%	551.66	153.73	59.91	9.99	1,120.39
		+20%	551.66	153.73	59.99	9.99	1,119.68
*b*_3_	10	−20%	551.66	153.73	59.99	9.99	1,118.8
		−10%	551.66	153.73	59.99	9.99	1,119.96
		+10%	554.05	120.026	59.99	9.99	1,090.3
		+20%	554.05	120.026	59.99	9.99	1,090.3
*β*	0.0003	−20%	551.68	131.41	59.9	9.99	1,099.89
		−10%	551.68	131.41	59.9	9.99	1,099.89
		+10%	551.68	131.41	59.9	9.99	1,099.89
		+20%	551.68	131.41	59.9	9.99	1,099.89
*g*_2_	3	−20%	551.68	131.41	59.9	9.99	1,099.89
		−10%	551.68	131.41	59.9	9.99	1,099.89
		+10%	551.68	131.41	59.9	9.99	1,099.89
		+20%	551.68	131.41	59.9	9.99	1,099.89
*g*_4_	8	−20%	552.93	151.2	59.99	9.99	1,119.3
		−10%	551.68	131.4	59.91	9.99	1,099.68
		+10%	551.68	131.4	59.91	9.99	1,099.68
		+20%	551.68	131.4	59.91	9.99	1,099.68
*ω*_1_	6	−20%	555.2	155.86	59.97	9.99	1,118.1
		−10%	554.4	163.4	59.99	9.99	1,128.59
		+10%	549.9	120.3	59.97	9.99	1,092
		+20%	597.57	120	59.99	9.99	1,131.8
*ω*_2_	10	−20%	551.3	154.7	59.97	9.97	1,114.9
		−10%	551.3	120	59.98	9.98	1,084.1
		+10%	554.1	120	59.99	9.99	1,092.1
		+20%	549.9	120.3	59.97	9.94	1,092.9

#### Inferences

• The change in the ordering cost by 10% and 20% causes the cycle time (*t*_1_), preservation investment (*ξ*) and total inventory-related cost to change in a downward direction. Conversely a 10% and 20% increase in *CC*_*O*_ results in a slower changing of these decision variables and these are the direct effects of ordering cost on the behavior of the system.
• Change in one of the parameters of preservation *q*, of the range of ±20%, produces no substantial changes in the average total cost of inventory or the variables of decision. This implies that the model is comparatively not sensitive to the change in preservation effectiveness in the existing operating conditions.
• It is observed that the parameter c of the deterioration rate produces a slight effect on the system results. The total inventory cost and decision variables do not change significantly with the change of c significantly by ±20%, indicating that the deterioration rate per se is not especially significant to the cost results in the modeled situation.
• Of all the parameters, the deterioration shape factor d poses a significant effect in the system. A reduction in d significantly increases the total inventory cost whereas the reverse is true. Here is where d is a parameter that is sensitive and one that directly influences efficiency of the system.
• The demand coefficient changes (*a*_3_ and *b*_3_) also have an impact on preservation investment and average total cost. Minimizing these parameters tends to reduce the total expenditure, and maximizing those raises the preservation investment and the average total cost, which implies that the demand patterns are crucial in the decisions of inventory.
• Changes in *β* and *g*_2_ have small effects and their variations can only be noticed at a very small scale (fourth decimal place), proving that the given parameters are not as sensitive in the analyzed context of this model.
• The changes in the penalty cost of lost sales slightly increase the total inventory spending, whereas the changes in *g*_4_ lead to the slight savings. This parameter is moderately sensitive of the cost outcomes.
• When *ω*_1_ and *ω*_2_ are changed to specify the phase changes in the trapezoidal demand pattern, one can observe significant but relatively insignificant changes in preservation investment and overall inventory cost. In spite of the fact the changes are not radical, progressive swings in performance attest to the fact that the presence of demand intervals is critical in determining inventory performance. In particular, a promotion or postponement of these breakpoints influences the nature of the interaction of demand intensity and inventory replenishment cycles, preservation strategies and cost optimization.

### Managerial insights

Routine strategies may not be sufficient in the livestock inventory. The suggested model reinvents an inventory planning to combine both biological reality and operational efficiency that can offer livestock enterprises an efficient roadmap to make improved decisions.

• **Harnessing biological dynamics for inventory timing:** Biological dynamics allow managers to understand when a product is best to sell and/or replace at the same time modeling the enhancement of livestock improvement and decay, so that the value of the product is captured before the degradation rate accelerates.
• **Stock movement and seasonal demand shifts:** A three-stage form of demand structure will be incorporated in order to make sure that a stock decision is made based on the actual market cycles rather than wasting resources in lean periods and maximizing it during peak periods.
• **Preservation spending as a strategic investment:** This model does not treat preservation as an expense, but instead, it repositioned it as an investment, with a direct negative impact on the rate of deterioration and livestock quality, particularly during a market price peak.
• **Smart holding cost management:** The system is aware of the fact that the cost of storage and care rises non-linearly and will therefore encourage the use of optimized cycle length to ensure continuity of service and minimization of cost.
• **Responsive shortage handling:** The approach is responsive in that by accommodating the concept of partial backlogging it represents in reality the response of consumers to stock outs wherein the managers have to balance the cost of lost sales versus delayed order fulfillment.
• **Optimization of data using modern algorithm:** With the help of GA and KHA optimizations, complicated managements such as price determination, investment levels, as well as replenishment scheduling are determined effectively, and thus efficiency is maintained in the long-run.
• **Parameter sensitivity as a control lever:** Results of the sensitivity analysis allow the managers to parameterize strategies in response to the impact of the crucial operational parameters, thus making them implement proactive changes to the evolving internal or external factors.

## Results

### Dynamics of inventory when the demand varies

These insights are supported by the use of graphical outputs. Figures 7, 8, 9, 10, 11, and 12 discuss the dynamics of the operations in all three scenarios, using visual data that demonstrates how the inventory can be modified with the change in demand phases and conditions of warehouses.

### Optimal inventory policies acquired with GA and KHA

The effectiveness of the suggested model is also supported by numerical outputs. Tables 2, 3, and 4 are aggregate results of optimal inventory policies identified through the GA and KHA algorithms, which can be used as a strategic plan by managers to optimize their ordering, preservation, and pricing policies.

### Sensitivity analysis of model parameters

Table 5 explores the direct impact of changing the operational parameters on the restructuring of inventory outcomes that results in a data-driven basis of adaptive decision-making.

### Response to parametric changes in inventory performance

Figure 13 converts the parameter changes into the graphical tendencies and shows the effects of the changes of ordering cost, deterioration rates, preservation investment, demand changes, and elements of holding costs on the inventory efficiency and cost behavior.

In general, this model is applicable as a pragmatic decision-support model, which facilitates livestock administrators to convert the biological fluctuation and market uncertainty into strategic chances of profitable operations that are sustainable.

#### Comparative and managerial implications

Table 5 results provide a number of practical suggestions regarding livestock inventory management in reality. It has been analyzed that the most significant impact on the overall cost of inventory is the control of deterioration, knowledge of demand changes, which emphasizes the need to invest in preservation and take timely decisions on replenishment. In contrast to the earlier inventory models that viewed deterioration and preservation independently, the model proposed unites the two phenomena with partial backlogging and cubic holding costs to maintain a better cost control and responsivity to the dynamics in the market. In addition, the sensitivity results match previous research results in deterioration-based inventory models, whereby the deterioration shape factor and the demand pattern parameters are predominant factors in influencing the policy of inventory. Nevertheless, our model goes beyond the existing body of work through that it includes time and price sensitive trapezoidal demand, and hence it can better match the production and sales cycle with the market trends. This holistic sensitivity analysis therefore does not only confirm the soundness of the model, but also offers viable management lessons on how best to develop adaptive, cost effective and sustainable livestock inventory procedures.

## Discussion

In this study, a detailed livestock inventory model combining major real life factors, including amelioration, deterioration, preservation investment, trapezoidal time and price dependent demand, cubic holding cost, and partial backlogging is proposed into a single study model. The model, through the integration of these factors that are interrelated, is effective in capturing the biological and economic aspects of livestock systems. The use of the GA and KHA guarantees effective global optimization of the decision variables such selling price, cycle time, preservation expenditure and overall cost; therefore, the strength and trustworthiness of the suggested method can be confirmed. The results indicate that preservation investment and dynamic pricing decisions have a significant positive impact on the performance of the systems by reducing the overall cost and increasing the profitability. This model adds a realistic decision support framework that balances efficiency in the operation with the sustainability goals and the livestock managers will have better control of costs, less wastage and more efficient use of resources. This combination of amelioration and deterioration processes further enables the contrasting inventory decisions to represent the biological facts and the market trends.

### Limitations of the study

The current study is limited to a single-item deterministic environment with replenishment in the instant, though. Others like stochastic demand, uncertain lead time and multi-product interactions have not been explored. In addition, although GA and KHA have good convergence properties, the computation cost might rise when used in a high-scale or multi-objective setting.

**Future research** can build on these and develop uncertainty in the form of stochastic or fuzzy demand modeling, multi-item and multi echelon supply chain structures, or trade credit policies that are dynamic. The discussion of carbon emission limitations, renewable preservation technologies, and machine learning-based forecasting might increase the flexibility and sustainability of the model even more. Simply, the article presents a solid platform on sustainable livestock inventory management as it cuts across the operational intricacy and environmental accountability. The suggested framework does not only contribute to the further theoretical comprehension of combined inventory systems, but it also offers practical solutions to the practice that lets practitioners strike the balance between economic and ecological success with livestock operations today.

## Resource availability

### Lead contact

Further information and requests for resources should be directed to and will be fulfilled by the lead contact. Dr. Umamaheswari S (umamaheswari.suk@vit.ac.in).

### Materials availability

This study did not generate new unique reagents or materials.

### Data and code availability

• Data sharing does not apply to this article as no new data were created or analyzed in this study.
• MATLAB codes used for the genetic algorithm (GA) and krill herd algorithm (KHA) optimization are available from the corresponding author upon reasonable request.
• Any additional information required to reanalyze the data reported in this article is available from the lead contact upon request.

## Acknowledgments

The authors would like to thank the 10.13039/100019904Vellore Institute of Technology, Chennai, India, for the infrastructural support provided to conduct this research.

## Author contributions

A.E.: conceptualization, methodology, data curation, formal analysis, software, validation, visualization, and writing – original draft. U.S.: conceptualization, methodology, software, supervision, validation, visualization, and writing – review and editing.

## Declaration of interests

The authors declare no competing interests.

## Declaration of generative AI and AI-assisted technologies in the writing process

The authors declare that ChatGPT (OpenAI) was used solely for language editing purposes, including grammar, clarity, and sentence structure improvements. No content generation, data analysis, interpretation, or scientific conclusions were influenced by the AI tool. The authors take full responsibility for the originality and accuracy of the manuscript.

## STAR★Methods

### Key resources table

**Table:** 

REAGENT or RESOURCE	SOURCE	IDENTIFIER
**Software and algorithms**		

MATLAB (R2023a)	MathWorks	https://scicrunch.org/resolver/SCR_001622
Genetic Algorithm (GA)	MathWorks, Global Optimization Toolbox	N/A
Krill Herd Algorithm (KHA)	This paper	N/A

**Deposited data**		

Numerical simulation data supporting optimization results	This paper	N/A

**Other**		

MATLAB scripts for GA and KHA optimization	This paper	Available upon reasonable request

### Method details

This study develops a deterministic mathematical inventory optimization model for livestock systems incorporating Weibull-based amelioration and deterioration, trapezoidal time and price-dependent demand, cubic holding cost, preservation investment, and partial backlogging. The total composite cost function is formulated analytically under different demand scenarios. Optimization of decision variables, including order quantity, preservation investment, selling price, and cycle time, is carried out using metaheuristic algorithms.

Mathematical Classification: 90B05, 68W50.

### Quantification and statistical analysis

All numerical computations and optimization procedures were performed using MATLAB. GA and KHA were employed to minimize the total composite cost function. Convergence behavior and solution robustness were evaluated through multiple simulation runs under different parameter settings.

## Appendix A

### Case 1

Ameliorating Cost

(Equation A.1) $${AA}_{c}=g_{1}\;\left(\frac{\psi e\left(m\left(\xi \right)-1\right)cfR_{1}^{f+d}}{f+d}+\frac{\psi e^{2}R_{1}^{2}f}{2}+\psi eR_{1}^{f}-\left(\frac{2e^{2}\left(d+2\right)f\left(\left(b_{1}d+b_{1}\right)f+b_{1}d+b_{1}\right)\left({t_{1}}^{2f+2}-{R_{1}}^{2f+2}\right)}{P^{J}\left(2f+2\right)\left(\left(2d^{2}+6d+4\right)f^{2}+\left(6d^{2}+18d+12\right)f+4d^{2}+12d+8\right)}+\frac{e^{2}\left(d+1\right)f\left(\left({2a}_{1}d+4a_{1}\right)f+4a_{1}d+8a_{1}\right)\left({t_{1}}^{2f+1}-{R_{1}}^{2f+1}\right)}{P^{J}\left(2f+1\right)\left(\left(2d^{2}+6d+4\right)f^{2}+\left(6d^{2}+18d+12\right)f+4d^{2}+12d+8\right)}+\frac{ecf\left(\left(2m\left(\xi \right)-2\right)f+4m\left(\xi \right)-4\right)\left(\left(b_{1}d+b_{1}\right)f+b_{1}d+b_{1}\right)\left({t_{1}}^{f+d+2}-{R_{1}}^{f+d+2}\right)}{P^{J}\left(f+d+2\right)\left(\left(2d^{2}+6d+4\right)f^{2}+\left(6d^{2}+18d+12\right)f+4d^{2}+12d+8\right)}+\frac{e\left(m\left(\xi \right)-1\right)cf\left(f+1\right)\left(\left({2a}_{1}d+4a_{1}\right)f+4a_{1}d+8a_{1}\right)\left({t_{1}}^{f+d+1}-{R_{1}}^{f+d+1}\right)}{P^{J}\left(f+d+1\right)\left(\left(2d^{2}+6d+4\right)f^{2}+\left(6d^{2}+18d+12\right)f+4d^{2}+12d+8\right)}+\frac{ef\left(\left(-d-2\right)f-2d-4\right)\left(\left(b_{1}d+b_{1}\right)f+b_{1}d+b_{1}\right)\left({t_{1}}^{f+2}-{R_{1}}^{f+2}\right)}{P^{J}\left(f+2\right)\left(\left(2d^{2}+6d+4\right)f^{2}+\left(6d^{2}+18d+12\right)f+4d^{2}+12d+8\right)}+\frac{ef\left(\left(-d-1\right)f-d-1\right)\left(\left({2a}_{1}d+4a_{1}\right)f+4a_{1}d+8a_{1}\right)\left({t_{1}}^{f+1}-{R_{1}}^{f+1}\right)}{P^{J}\left(f+1\right)\left(\left(2d^{2}+6d+4\right)f^{2}+\left(6d^{2}+18d+12\right)f+4d^{2}+12d+8\right)}+\frac{e\left(\left({2a}_{1}d+4a_{1}\right)f+4a_{1}d+8a_{1}\right)\left(\left(d+1\right)ft_{1}+dt_{1}+t_{1}-e\left(d+1\right){t_{1}}^{f+1}-\left(m\left(\xi \right)-1\right)c{t_{1}}^{d+1}\left(f+1\right)\right)\left({t_{1}}^{f}-{R_{1}}^{f}\right)}{P^{J}\left(\left(2d^{2}+6d+4\right)f^{2}+\left(6d^{2}+18d+12\right)f+4d^{2}+12d+8\right)}+\frac{e\left(\left(b_{1}d+b_{1}\right)f+b_{1}d+b_{1}\right)\left(-2e\left(d+2\right){t_{1}}^{f+2}-\left(\left(-d-2\right)f-2d-4\right){t_{1}}^{2}-c{t_{1}}^{d+2}\left(\left(2m\left(\xi \right)-2\right)f+4m\left(\xi \right)-4\right)\right)\left({t_{1}}^{f}-{R_{1}}^{f}\right)}{P^{J}\left(\left(2d^{2}+6d+4\right)f^{2}+\left(6d^{2}+18d+12\right)f+4d^{2}+12d+8\right)}\right)\right)$$

Deteriorating Cost

(Equation A.2) $${DD}_{c}=g_{2}\;\left(-\frac{\psi \left(m\left(\xi \right)-1\right)cR_{1}^{d}\left(2edR_{1}^{f}+\left(m\left(\xi \right)-1\right)c\left(f+d\right)R_{1}^{d}-2f-2d\right)fR_{1}^{f+d}}{2\left(f+d\right)}-\left(\frac{2e\left(1-m\left(\xi \right)\right)cd\left(d+2\right)\left(\left(b_{1}d+b_{1}\right)f+b_{1}d+b_{1}\right)\left({t_{1}}^{f+d+2}-{R_{1}}^{f+d+2}\right)}{P^{J}\left(f+d+2\right)\left(\left(2d^{2}+6d+4\right)f^{2}+\left(6d^{2}+18d+12\right)f+4d^{2}+12d+8\right)}+\frac{e\left(1-m\left(\xi \right)\right)cd\left(d+1\right)\left(\left({2a}_{1}d+4a_{1}\right)f+4a_{1}d+8a_{1}\right)\left({t_{1}}^{f+d+1}-{R_{1}}^{f+d+1}\right)}{P^{J}\left(f+d+1\right)\left(\left(2d^{2}+6d+4\right)f^{2}+\left(6d^{2}+18d+12\right)f+4d^{2}+12d+8\right)}+\frac{c^{2}\left(1-m\left(\xi \right)\right)d\left(\left(2m\left(\xi \right)-2\right)f+4m\left(\xi \right)-4\right)\left(\left(b_{1}d+b_{1}\right)f+b_{1}d+b_{1}\right)\left({t_{1}}^{2d+2}-{R_{1}}^{2d+2}\right)}{P^{J}\left(2d+2\right)\left(\left(2d^{2}+6d+4\right)f^{2}+\left(6d^{2}+18d+12\right)f+4d^{2}+12d+8\right)}+\frac{c^{2}\left(1-m\left(\xi \right)\right)\left(m\left(\xi \right)-1\right)d\left(f+1\right)\left(\left({2a}_{1}d+4a_{1}\right)f+4a_{1}d+8a_{1}\right)\left({t_{1}}^{2d+1}-{R_{1}}^{2d+1}\right)}{P^{J}\left(2d+1\right)\left(\left(2d^{2}+6d+4\right)f^{2}+\left(6d^{2}+18d+12\right)f+4d^{2}+12d+8\right)}+\frac{\left(1-m\left(\xi \right)\right)cd\left(\left(-d-2\right)f-2d-4\right)\left(\left(b_{1}d+b_{1}\right)f+b_{1}d+b_{1}\right)\left({t_{1}}^{d+2}-{R_{1}}^{d+2}\right)}{P^{J}\left(d+2\right)\left(\left(2d^{2}+6d+4\right)f^{2}+\left(6d^{2}+18d+12\right)f+4d^{2}+12d+8\right)}+\frac{\left(1-m\left(\xi \right)\right)cd\left(\left(-d-1\right)f-d-1\right)\left(\left({2a}_{1}d+4a_{1}\right)f+4a_{1}d+8a_{1}\right)\left({t_{1}}^{d+1}-{R_{1}}^{d+1}\right)}{P^{J}\left(d+1\right)\left(\left(2d^{2}+6d+4\right)f^{2}+\left(6d^{2}+18d+12\right)f+4d^{2}+12d+8\right)}+\frac{\left(1-m\left(\xi \right)\right)c\left(\left({2a}_{1}d+4a_{1}\right)f+4a_{1}d+8a_{1}\right)\left(\left(d+1\right)ft_{1}+dt_{1}+t_{1}-e\left(d+1\right){t_{1}}^{f+1}-\left(m\left(\xi \right)-1\right)c{t_{1}}^{d+1}\left(f+1\right)\right)\left({t_{1}}^{d}-{R_{1}}^{d}\right)}{P^{J}\left(\left(2d^{2}+6d+4\right)f^{2}+\left(6d^{2}+18d+12\right)f+4d^{2}+12d+8\right)}+\frac{\left(1-m\left(\xi \right)\right)c\left(\left(b_{1}d+b_{1}\right)f+b_{1}d+b_{1}\right)\left(-2e\left(d+2\right){t_{1}}^{f+2}-\left(\left(-d-2\right)f-2d-4\right){t_{1}}^{2}-c{t_{1}}^{d+2}\left(\left(2m\left(\xi \right)-2\right)f+4m\left(\xi \right)-4\right)\right)\left({t_{1}}^{d}-{R_{1}}^{d}\right)}{P^{J}\left(\left(2d^{2}+6d+4\right)f^{2}+\left(6d^{2}+18d+12\right)f+4d^{2}+12d+8\right)}\right)\right)$$

Holding Cost

(Equation A.3) $${HH}_{c}=\frac{e\psi \beta R_{1}^{f+4}}{f+4}+\frac{e\psi \alpha R_{1}^{f+2}}{f+2}+\frac{\psi \left(m\left(\xi \right)-1\right)c\beta R_{1}^{d+4}}{d+4}+\frac{\psi \left(m\left(\xi \right)-1\right)c\alpha R_{1}^{d+2}}{d+2}+\frac{\psi \beta R_{1}^{4}+2\psi \alpha R_{1}^{2}}{d+2}-\left(\frac{2e\left(d+2\right)\left(\left(b_{1}d+b_{1}\right)f+b_{1}d+b_{1}\right)\beta \left({t_{1}}^{f+6}-{R_{1}}^{f+6}\right)}{P^{J}\left(f+6\right)\left(\left(2d^{2}+6d+4\right)f^{2}+\left(6d^{2}+18d+12\right)f+4d^{2}+12d+8\right)}+\frac{e\left(d+1\right)\left(\left({2a}_{1}d+4a_{1}\right)f+4a_{1}d+8a_{1}\right)\beta \left({t_{1}}^{f+5}-{R_{1}}^{f+5}\right)}{P^{J}\left(f+5\right)\left(\left(2d^{2}+6d+4\right)f^{2}+\left(6d^{2}+18d+12\right)f+4d^{2}+12d+8\right)}+\frac{2e\left(d+2\right)\left(\left(b_{1}d+b_{1}\right)f+b_{1}d+b_{1}\right)\alpha \left({t_{1}}^{f+4}-{R_{1}}^{f+4}\right)}{P^{J}\left(f+4\right)\left(\left(2d^{2}+6d+4\right)f^{2}+\left(6d^{2}+18d+12\right)f+4d^{2}+12d+8\right)}+\frac{e\left(d+1\right)\left(\left({2a}_{1}d+4a_{1}\right)f+4a_{1}d+8a_{1}\right)\alpha \left({t_{1}}^{f+3}-{R_{1}}^{f+3}\right)}{P^{J}\left(f+3\right)\left(\left(2d^{2}+6d+4\right)f^{2}+\left(6d^{2}+18d+12\right)f+4d^{2}+12d+8\right)}+\frac{c\left(\left(2m\left(\xi \right)-2\right)f+4m\left(\xi \right)-4\right)\left(\left(b_{1}d+b_{1}\right)f+b_{1}d+b_{1}\right)\beta \left({t_{1}}^{d+6}-{R_{1}}^{d+6}\right)}{P^{J}\left(d+6\right)\left(\left(2d^{2}+6d+4\right)f^{2}+\left(6d^{2}+18d+12\right)f+4d^{2}+12d+8\right)}+\frac{\left(m\left(\xi \right)-1\right)c\left(f+1\right)\left(\left({2a}_{1}d+4a_{1}\right)f+4a_{1}d+8a_{1}\right)\beta \left({t_{1}}^{d+5}-{R_{1}}^{d+5}\right)}{P^{J}\left(d+5\right)\left(\left(2d^{2}+6d+4\right)f^{2}+\left(6d^{2}+18d+12\right)f+4d^{2}+12d+8\right)}+\frac{c\left(\left(2m\left(\xi \right)-2\right)f+4m\left(\xi \right)-4\right)\left(\left(b_{1}d+b_{1}\right)f+b_{1}d+b_{1}\right)\alpha \left({t_{1}}^{d+4}-{R_{1}}^{d+4}\right)}{P^{J}\left(d+4\right)\left(\left(2d^{2}+6d+4\right)f^{2}+\left(6d^{2}+18d+12\right)f+4d^{2}+12d+8\right)}+\frac{\left(m\left(\xi \right)-1\right)c\left(f+1\right)\left(\left({2a}_{1}d+4a_{1}\right)f+4a_{1}d+8a_{1}\right)\alpha \left({t_{1}}^{d+3}-{R_{1}}^{d+3}\right)}{P^{J}\left(d+3\right)\left(\left(2d^{2}+6d+4\right)f^{2}+\left(6d^{2}+18d+12\right)f+4d^{2}+12d+8\right)}+\frac{\left(\left(-d-2\right)f-2d-4\right)\left(\left(b_{1}d+b_{1}\right)f+b_{1}d+b_{1}\right)\beta \left({t_{1}}^{6}-{R_{1}}^{6}\right)}{P^{J}6\left(\left(2d^{2}+6d+4\right)f^{2}+\left(6d^{2}+18d+12\right)f+4d^{2}+12d+8\right)}+\frac{\left(\left(-d-1\right)f-d-1\right)\left(\left({2a}_{1}d+4a_{1}\right)f+4a_{1}d+8a_{1}\right)\beta \left({t_{1}}^{5}-{R_{1}}^{5}\right)}{P^{J}5\left(\left(2d^{2}+6d+4\right)f^{2}+\left(6d^{2}+18d+12\right)f+4d^{2}+12d+8\right)}+\frac{\beta \left(\left({2a}_{1}d+4a_{1}\right)f+4a_{1}d+8a_{1}\right)\left(\left(d+1\right)ft_{1}+dt_{1}+t_{1}-e\left(d+1\right){t_{1}}^{f+1}-\left(m\left(\xi \right)-1\right)c{t_{1}}^{d+1}\left(f+1\right)\right)\left({t_{1}}^{4}-{R_{1}}^{4}\right)}{P^{J}4\left(\left(2d^{2}+6d+4\right)f^{2}+\left(6d^{2}+18d+12\right)f+4d^{2}+12d+8\right)}+\frac{\beta \left(\left(b_{1}d+b_{1}\right)f+b_{1}d+b_{1}\right)\left(-2e\left(d+2\right){t_{1}}^{f+2}-\left(\left(-d-2\right)f-2d-4\right){t_{1}}^{2}-c{t_{1}}^{d+2}\left(\left(2m\left(\xi \right)-2\right)f+4m\left(\xi \right)-4\right)\right)\left({t_{1}}^{4}-{R_{1}}^{4}\right)}{P^{J}4\left(\left(2d^{2}+6d+4\right)f^{2}+\left(6d^{2}+18d+12\right)f+4d^{2}+12d+8\right)}+\frac{\beta \left(\alpha \left(-d-2\right)f-2d-4\right)\left(\left(b_{1}d+b_{1}\right)f+b_{1}d+b_{1}\right)\left({t_{1}}^{4}-{R_{1}}^{4}\right)}{P^{J}4\left(\left(2d^{2}+6d+4\right)f^{2}+\left(6d^{2}+18d+12\right)f+4d^{2}+12d+8\right)}+\frac{\left(\left(-d-1\right)f-d-1\right)\left(\left({2a}_{1}d+4a_{1}\right)f+4a_{1}d+8a_{1}\right)\alpha \left({t_{1}}^{3}-{R_{1}}^{3}\right)}{P^{J}3\left(\left(2d^{2}+6d+4\right)f^{2}+\left(6d^{2}+18d+12\right)f+4d^{2}+12d+8\right)}+\frac{\alpha \left(\left({2a}_{1}d+4a_{1}\right)f+4a_{1}d+8a_{1}\right)\left(\left(d+1\right)ft_{1}+dt_{1}+t_{1}-e\left(d+1\right){t_{1}}^{f+1}-\left(m\left(\xi \right)-1\right)c{t_{1}}^{d+1}\left(f+1\right)\right)\left({t_{1}}^{2}-{R_{1}}^{2}\right)}{P^{J}2\left(\left(2d^{2}+6d+4\right)f^{2}+\left(6d^{2}+18d+12\right)f+4d^{2}+12d+8\right)}+\frac{\alpha \left(\left(b_{1}d+b_{1}\right)f+b_{1}d+b_{1}\right)\left(-2e\left(d+2\right){t_{1}}^{f+2}-\left(\left(-d-2\right)f-2d-4\right){t_{1}}^{2}-c{t_{1}}^{d+2}\left(\left(2m\left(\xi \right)-2\right)f+4m\left(\xi \right)-4\right)\right)\left({t_{1}}^{2}-{R_{1}}^{2}\right)}{P^{J}2\left(\left(2d^{2}+6d+4\right)f^{2}+\left(6d^{2}+18d+12\right)f+4d^{2}+12d+8\right)}\right)$$

Backorder Cost

(Equation A.4) $$T_{BB}=-g_{3}\left(\frac{b_{1}\left(\delta \left(\left(\omega_{1}-t_{1}\right)-t_{1}\right)-2\right)e^{\delta \left(\omega_{1}-t_{1}\right)-\delta \left(t_{1}+T\right)}}{\delta^{3}P^{J}}-\frac{a_{1}\left(\omega_{1}-t_{1}\right)}{{\delta P}^{J}}-\frac{e^{-\delta T}\left(a_{2}+b_{2}T\right)\left(e^{\delta \left(\omega_{2}-\omega_{1}\right)}-\delta e^{\delta \omega_{1}\left(\omega_{2}-\omega_{1}\right)}\right)}{\delta^{2}P^{J}}-\frac{\left(b_{1}\left(\delta \left(\omega_{1}-t_{1}\right)+\delta a_{1}-1\right)e^{\delta \left(\omega_{1}-t_{1}\right)-T}\right)\left(\omega_{2}-\omega_{1}\right)}{\delta^{2}P^{J}}+\frac{\left(b_{3}\left(\delta \left(\left(T-\omega_{2}\right)-\omega_{2}\right)-1-a_{3}\delta -1\right)e^{\delta \left(\left(T-\omega_{2}\right)-\omega_{2}\right)-T}\right)}{\delta^{3}P^{J}}+\frac{\left(a_{2}+b_{2}T\right)\left(e^{\delta \left(\omega_{1}-T\right)}-e^{\delta }\left(\omega_{2}-T\right)\right)\left(T-\omega_{2}\right)}{\delta P^{J}}-\frac{\left(b_{1}\left(\delta \left(\omega_{1}-t_{1}\right)+\delta a_{1}-1\right)e^{\delta \left(\omega_{1}-t_{1}\right)-T}\right)\left(T-\omega_{2}\right)}{\delta^{2}P^{J}}\right)$$

Cost of Lost Sales

(Equation A.5) $$T_{LL}=g_{4}\left(\frac{a_{1}\left(\omega_{1}-t_{1}\right)}{P^{J}}+\frac{b_{1}\left({\omega_{1}}^{2}-{t_{1}}^{2}\right)}{{2P}^{J}}-\frac{\left(b_{1}\left(\delta \left(\omega_{1}-t_{1}\right)-1\right)+\delta a_{1}\right)e^{\delta \left(\left(\omega_{1}-t_{1}\right)-T\right)}}{\delta^{2}P^{J}}+\frac{\left(a_{2}+b_{2}T\right)\left(\omega_{2}-\omega_{1}\right)}{P^{J}}-\frac{\left(a_{2}+b_{2}T\right)e^{\delta \left(\left(\omega_{2}-\omega_{1}\right)-T\right)}}{\delta P^{J}}+\frac{a_{3}\left(T-\omega_{2}\right)}{P^{J}}+\frac{b_{3}\left({T^{2}-\omega_{2}}^{2}\right)}{{2P}^{J}}-\frac{\left(b_{3}\left(\delta \left(T-\omega_{2}\right)-1\right)+\delta a_{3}\right)e^{\delta \left(\left(T-\omega_{2}\right)-T\right)}}{\delta^{2}P^{J}}\right)$$

### Case 2

Ameliorating cost

(Equation A.6) $${AA}_{c}=g_{1}\;\left(\frac{\psi e\left(m\left(\xi \right)-1\right)cfR_{1}^{f+d}}{f+d}+\frac{\psi e^{2}R_{1}^{2}f}{2}+\psi eR_{1}^{f}-\left(\frac{2e^{2}\left(d+2\right)f\left(\left(b_{1}d+b_{1}\right)f+b_{1}d+b_{1}\right)\left({\omega_{1}}^{2f+2}-{R_{1}}^{2f+2}\right)}{P^{J}\left(2f+2\right)\left(\left(2d^{2}+6d+4\right)f^{2}+\left(6d^{2}+18d+12\right)f+4d^{2}+12d+8\right)}+\frac{e^{2}\left(d+1\right)f\left(\left({2a}_{1}d+4a_{1}\right)f+4a_{1}d+8a_{1}\right)\left({\omega_{1}}^{2f+1}-{R_{1}}^{2f+1}\right)}{P^{J}\left(2f+1\right)\left(\left(2d^{2}+6d+4\right)f^{2}+\left(6d^{2}+18d+12\right)f+4d^{2}+12d+8\right)}+\frac{ecf\left(\left(2m\left(\xi \right)-2\right)f+4m\left(\xi \right)-4\right)\left(\left(b_{1}d+b_{1}\right)f+b_{1}d+b_{1}\right)\left({\omega_{1}}^{f+d+2}-{R_{1}}^{f+d+2}\right)}{P^{J}\left(f+d+2\right)\left(\left(2d^{2}+6d+4\right)f^{2}+\left(6d^{2}+18d+12\right)f+4d^{2}+12d+8\right)}+\frac{e\left(m\left(\xi \right)-1\right)cf\left(f+1\right)\left(\left({2a}_{1}d+4a_{1}\right)f+4a_{1}d+8a_{1}\right)\left({\omega_{1}}^{f+d+1}-{R_{1}}^{f+d+1}\right)}{P^{J}\left(f+d+1\right)\left(\left(2d^{2}+6d+4\right)f^{2}+\left(6d^{2}+18d+12\right)f+4d^{2}+12d+8\right)}+\frac{ef\left(\left(-d-2\right)f-2d-4\right)\left(\left(b_{1}d+b_{1}\right)f+b_{1}d+b_{1}\right)\left({\omega_{1}}^{f+2}-{R_{1}}^{f+2}\right)}{P^{J}\left(f+2\right)\left(\left(2d^{2}+6d+4\right)f^{2}+\left(6d^{2}+18d+12\right)f+4d^{2}+12d+8\right)}+\frac{ef\left(\left(-d-1\right)f-d-1\right)\left(\left({2a}_{1}d+4a_{1}\right)f+4a_{1}d+8a_{1}\right)\left({\omega_{1}}^{f+1}-{R_{1}}^{f+1}\right)}{P^{J}\left(f+1\right)\left(\left(2d^{2}+6d+4\right)f^{2}+\left(6d^{2}+18d+12\right)f+4d^{2}+12d+8\right)}+\frac{e\left(\left({2a}_{1}d+4a_{1}\right)f+4a_{1}d+8a_{1}\right)\left(\left(d+1\right)f\omega_{1}+d\omega_{1}+\omega_{1}-e\left(d+1\right){\omega_{1}}^{f+1}-\left(m\left(\xi \right)-1\right)c{\omega_{1}}^{d+1}\left(f+1\right)\right)\left({\omega_{1}}^{f}-{R_{1}}^{f}\right)}{P^{J}\left(\left(2d^{2}+6d+4\right)f^{2}+\left(6d^{2}+18d+12\right)f+4d^{2}+12d+8\right)}+\frac{e\left(\left(b_{1}d+b_{1}\right)f+b_{1}d+b_{1}\right)\left(-2e\left(d+2\right){\omega_{1}}^{f+2}-\left(\left(-d-2\right)f-2d-4\right){\omega_{1}}^{2}-c{\omega_{1}}^{d+2}\left(\left(2m\left(\xi \right)-2\right)f+4m\left(\xi \right)-4\right)\right)\left({\omega_{1}}^{f}-{R_{1}}^{f}\right)}{P^{J}\left(\left(2d^{2}+6d+4\right)f^{2}+\left(6d^{2}+18d+12\right)f+4d^{2}+12d+8\right)}\right)+\frac{e\left(a_{2}+b_{2}T\right)}{P^{J}}\left(\frac{\left(m\left(\xi \right)-1\right)c\left({t_{1}}^{d+1}-{\omega_{1}}^{d+1}\right)}{d+1}+\frac{e\left({t_{1}}^{f+1}-{\omega_{1}}^{f+1}\right)}{f+1}+\left(t_{1}-\omega_{1}\right)\right)\left({\omega_{1}}^{f}-{R_{1}}^{f}\right)-\left(\frac{e^{2}\left(a_{2}+b_{2}T\right)\left(d+1\right)f\left({t_{1}}^{2f+1}-{\omega_{1}}^{2f+1}\right)}{P^{J}\left(2f+1\right)\left(\left(d+1\right)f+d+1\right)}+\frac{e\left(m\left(\xi \right)-1\right)\left(a_{2}+b_{2}T\right)\left(f+1\right)cf\left({t_{1}}^{f+d+1}-{\omega_{1}}^{f+d+1}\right)}{P^{J}\left(f+d+1\right)\left(\left(d+1\right)f+d+1\right)}+\frac{e\left(a_{2}+b_{2}T\right)\left(\left(-d-1\right)f-d-1\right)f\left({t_{1}}^{f+1}-{\omega_{1}}^{f+1}\right)}{P^{J}\left(f+1\right)\left(\left(d+1\right)f+d+1\right)}+\frac{e\left(a_{2}+b_{2}T\right)\left(\left(d+1\right)ft_{1}+dt_{1}+t_{1}-e\left(d+1\right){t_{1}}^{f+1}-\left(m\left(\xi \right)-1\right)c{t_{1}}^{d+1}\left(f+1\right)\right)\left({t_{1}}^{f}-{\omega_{1}}^{f}\right)}{P^{J}\left(\left(d+1\right)f+d+1\right)}\right)\right)$$

Deteriorating Cost

(Equation A.7) $${DD}_{c}=g_{2}\;\left(-\frac{\psi \left(m\left(\xi \right)-1\right)cR_{1}^{d}\left(2edR_{1}^{f}+\left(m\left(\xi \right)-1\right)c\left(f+d\right)R_{1}^{d}-2f-2d\right)fR_{1}^{f+d}}{2\left(f+d\right)}-\left(\frac{2e\left(1-m\left(\xi \right)\right)cd\left(d+2\right)\left(\left(b_{1}d+b_{1}\right)f+b_{1}d+b_{1}\right)\left({\omega_{1}}^{f+d+2}-{R_{1}}^{f+d+2}\right)}{P^{J}\left(f+d+2\right)\left(\left(2d^{2}+6d+4\right)f^{2}+\left(6d^{2}+18d+12\right)f+4d^{2}+12d+8\right)}+\frac{e\left(1-m\left(\xi \right)\right)cd\left(d+1\right)\left(\left({2a}_{1}d+4a_{1}\right)f+4a_{1}d+8a_{1}\right)\left({\omega_{1}}^{f+d+1}-{R_{1}}^{f+d+1}\right)}{P^{J}\left(f+d+1\right)\left(\left(2d^{2}+6d+4\right)f^{2}+\left(6d^{2}+18d+12\right)f+4d^{2}+12d+8\right)}+\frac{c^{2}\left(1-m\left(\xi \right)\right)d\left(\left(2m\left(\xi \right)-2\right)f+4m\left(\xi \right)-4\right)\left(\left(b_{1}d+b_{1}\right)f+b_{1}d+b_{1}\right)\left({\omega_{1}}^{2d+2}-{R_{1}}^{2d+2}\right)}{P^{J}\left(2d+2\right)\left(\left(2d^{2}+6d+4\right)f^{2}+\left(6d^{2}+18d+12\right)f+4d^{2}+12d+8\right)}+\frac{c^{2}\left(1-m\left(\xi \right)\right)\left(m\left(\xi \right)-1\right)d\left(f+1\right)\left(\left({2a}_{1}d+4a_{1}\right)f+4a_{1}d+8a_{1}\right)\left({\omega_{1}}^{2d+1}-{R_{1}}^{2d+1}\right)}{P^{J}\left(2d+1\right)\left(\left(2d^{2}+6d+4\right)f^{2}+\left(6d^{2}+18d+12\right)f+4d^{2}+12d+8\right)}+\frac{\left(1-m\left(\xi \right)\right)cd\left(\left(-d-2\right)f-2d-4\right)\left(\left(b_{1}d+b_{1}\right)f+b_{1}d+b_{1}\right)\left({\omega_{1}}^{d+2}-{R_{1}}^{d+2}\right)}{P^{J}\left(d+2\right)\left(\left(2d^{2}+6d+4\right)f^{2}+\left(6d^{2}+18d+12\right)f+4d^{2}+12d+8\right)}+\frac{\left(1-m\left(\xi \right)\right)cd\left(\left(-d-1\right)f-d-1\right)\left(\left({2a}_{1}d+4a_{1}\right)f+4a_{1}d+8a_{1}\right)\left({\omega_{1}}^{d+1}-{R_{1}}^{d+1}\right)}{P^{J}\left(d+1\right)\left(\left(2d^{2}+6d+4\right)f^{2}+\left(6d^{2}+18d+12\right)f+4d^{2}+12d+8\right)}+\frac{\left(1-m\left(\xi \right)\right)c\left(\left({2a}_{1}d+4a_{1}\right)f+4a_{1}d+8a_{1}\right)\left(\left(d+1\right)f\omega_{1}+d\omega_{1}+\omega_{1}-e\left(d+1\right){\omega_{1}}^{f+1}-\left(m\left(\xi \right)-1\right)c{\omega_{1}}^{d+1}\left(f+1\right)\right)\left({\omega_{1}}^{d}-{R_{1}}^{d}\right)}{P^{J}\left(\left(2d^{2}+6d+4\right)f^{2}+\left(6d^{2}+18d+12\right)f+4d^{2}+12d+8\right)}+\frac{\left(1-m\left(\xi \right)\right)c\left(\left(b_{1}d+b_{1}\right)f+b_{1}d+b_{1}\right)\left(-2e\left(d+2\right){\omega_{1}}^{f+2}-\left(\left(-d-2\right)f-2d-4\right){\omega_{1}}^{2}-c{\omega_{1}}^{d+2}\left(\left(2m\left(\xi \right)-2\right)f+4m\left(\xi \right)-4\right)\right)\left({\omega_{1}}^{d}-{R_{1}}^{d}\right)}{P^{J}\left(\left(2d^{2}+6d+4\right)f^{2}+\left(6d^{2}+18d+12\right)f+4d^{2}+12d+8\right)}\right)+\frac{\left(m\left(\xi \right)-1\right)a_{2}+b_{2}T}{P^{J}}\left(\frac{\left(m\left(\xi \right)-1\right)c\left({t_{1}}^{d+1}-{\omega_{1}}^{d+1}\right)}{d+1}+\frac{e\left({t_{1}}^{f+1}-{\omega_{1}}^{f+1}\right)}{f+1}+\left(t_{1}-\omega_{1}\right)\right)\left({\omega_{1}}^{d}-{R_{1}}^{d}\right)-\left(\frac{c^{2}d\left(1-m\left(\xi \right)\right)\left(m\left(\xi \right)-1\right)\left(a_{2}+b_{2}T\right)\left(f+1\right)f\left({t_{1}}^{2d+1}-{\omega_{1}}^{2d+1}\right)}{P^{J}\left(2d+1\right)\left(\left(d+1\right)f+d+1\right)}+\frac{e\left(m\left(\xi \right)-1\right)\left(a_{2}+b_{2}T\right)\left(d+1\right)cd\left({t_{1}}^{f+d+1}-{\omega_{1}}^{f+d+1}\right)}{P^{J}\left(f+d+1\right)\left(\left(d+1\right)f+d+1\right)}+\frac{\left(1-m\left(\xi \right)\right)cd\left(a_{2}+b_{2}T\right)\left(\left(-d-1\right)f-d-1\right)\left({t_{1}}^{d+1}-{\omega_{1}}^{d+1}\right)}{P^{J}\left(d+1\right)\left(\left(d+1\right)f+d+1\right)}+\frac{\left(1-m\left(\xi \right)\right)c\left(a_{2}+b_{2}T\right)\left(\left(d+1\right)ft_{1}+dt_{1}+t_{1}-e\left(d+1\right){t_{1}}^{f+1}-\left(m\left(\xi \right)-1\right)c{t_{1}}^{d+1}\left(f+1\right)\right)\left({t_{1}}^{d}-{\omega_{1}}^{d}\right)}{P^{J}\left(\left(d+1\right)f+d+1\right)}\right)\right)$$

Holding Cost

(Equation A.8) $${HH}_{c}=\frac{e\psi \beta R_{1}^{f+4}}{f+4}+\frac{e\psi \alpha R_{1}^{f+2}}{f+2}+\frac{\psi \left(m\left(\xi \right)-1\right)c\beta R_{1}^{d+4}}{d+4}+\frac{\psi \left(m\left(\xi \right)-1\right)c\alpha R_{1}^{d+2}}{d+2}+\frac{\psi \beta R_{1}^{4}+2\psi \alpha R_{1}^{2}}{d+2}-\left(\frac{2e\left(d+2\right)\left(\left(b_{1}d+b_{1}\right)f+b_{1}d+b_{1}\right)\beta \left({\omega_{1}}^{f+6}-{R_{1}}^{f+6}\right)}{P^{J}\left(f+6\right)\left(\left(2d^{2}+6d+4\right)f^{2}+\left(6d^{2}+18d+12\right)f+4d^{2}+12d+8\right)}+\frac{e\left(d+1\right)\left(\left({2a}_{1}d+4a_{1}\right)f+4a_{1}d+8a_{1}\right)\beta \left({\omega_{1}}^{f+5}-{R_{1}}^{f+5}\right)}{P^{J}\left(f+5\right)\left(\left(2d^{2}+6d+4\right)f^{2}+\left(6d^{2}+18d+12\right)f+4d^{2}+12d+8\right)}+\frac{2e\left(d+2\right)\left(\left(b_{1}d+b_{1}\right)f+b_{1}d+b_{1}\right)\alpha \left({\omega_{1}}^{f+4}-{R_{1}}^{f+4}\right)}{P^{J}\left(f+4\right)\left(\left(2d^{2}+6d+4\right)f^{2}+\left(6d^{2}+18d+12\right)f+4d^{2}+12d+8\right)}+\frac{e\left(d+1\right)\left(\left({2a}_{1}d+4a_{1}\right)f+4a_{1}d+8a_{1}\right)\alpha \left({\omega_{1}}^{f+3}-{R_{1}}^{f+3}\right)}{P^{J}\left(f+3\right)\left(\left(2d^{2}+6d+4\right)f^{2}+\left(6d^{2}+18d+12\right)f+4d^{2}+12d+8\right)}+\frac{c\left(\left(2m\left(\xi \right)-2\right)f+4m\left(\xi \right)-4\right)\left(\left(b_{1}d+b_{1}\right)f+b_{1}d+b_{1}\right)\beta \left({\omega_{1}}^{d+6}-{R_{1}}^{d+6}\right)}{P^{J}\left(d+6\right)\left(\left(2d^{2}+6d+4\right)f^{2}+\left(6d^{2}+18d+12\right)f+4d^{2}+12d+8\right)}+\frac{\left(m\left(\xi \right)-1\right)c\left(f+1\right)\left(\left({2a}_{1}d+4a_{1}\right)f+4a_{1}d+8a_{1}\right)\beta \left({\omega_{1}}^{d+5}-{R_{1}}^{d+5}\right)}{P^{J}\left(d+5\right)\left(\left(2d^{2}+6d+4\right)f^{2}+\left(6d^{2}+18d+12\right)f+4d^{2}+12d+8\right)}+\frac{c\left(\left(2m\left(\xi \right)-2\right)f+4m\left(\xi \right)-4\right)\left(\left(b_{1}d+b_{1}\right)f+b_{1}d+b_{1}\right)\alpha \left({\omega_{1}}^{d+4}-{R_{1}}^{d+4}\right)}{P^{J}\left(d+4\right)\left(\left(2d^{2}+6d+4\right)f^{2}+\left(6d^{2}+18d+12\right)f+4d^{2}+12d+8\right)}+\frac{\left(m\left(\xi \right)-1\right)c\left(f+1\right)\left(\left({2a}_{1}d+4a_{1}\right)f+4a_{1}d+8a_{1}\right)\alpha \left({\omega_{1}}^{d+3}-{R_{1}}^{d+3}\right)}{P^{J}\left(d+3\right)\left(\left(2d^{2}+6d+4\right)f^{2}+\left(6d^{2}+18d+12\right)f+4d^{2}+12d+8\right)}+\frac{\left(\left(-d-2\right)f-2d-4\right)\left(\left(b_{1}d+b_{1}\right)f+b_{1}d+b_{1}\right)\beta \left({\omega_{1}}^{6}-{R_{1}}^{6}\right)}{P^{J}6\left(\left(2d^{2}+6d+4\right)f^{2}+\left(6d^{2}+18d+12\right)f+4d^{2}+12d+8\right)}+\frac{\left(\left(-d-1\right)f-d-1\right)\left(\left({2a}_{1}d+4a_{1}\right)f+4a_{1}d+8a_{1}\right)\beta \left({\omega_{1}}^{5}-{R_{1}}^{5}\right)}{P^{J}5\left(\left(2d^{2}+6d+4\right)f^{2}+\left(6d^{2}+18d+12\right)f+4d^{2}+12d+8\right)}+\frac{\beta \left(\left({2a}_{1}d+4a_{1}\right)f+4a_{1}d+8a_{1}\right)\left(\left(d+1\right)f\omega_{1}+d\omega_{1}+\omega_{1}-e\left(d+1\right){\omega_{1}}^{f+1}-\left(m\left(\xi \right)-1\right)c{\omega_{1}}^{d+1}\left(f+1\right)\right)\left({\omega_{1}}^{4}-{R_{1}}^{4}\right)}{P^{J}4\left(\left(2d^{2}+6d+4\right)f^{2}+\left(6d^{2}+18d+12\right)f+4d^{2}+12d+8\right)}+\frac{\beta \left(\left(b_{1}d+b_{1}\right)f+b_{1}d+b_{1}\right)\left(-2e\left(d+2\right){\omega_{1}}^{f+2}-\left(\left(-d-2\right)f-2d-4\right){\omega_{1}}^{2}-c{\omega_{1}}^{d+2}\left(\left(2m\left(\xi \right)-2\right)f+4m\left(\xi \right)-4\right)\right)\left({\omega_{1}}^{4}-{R_{1}}^{4}\right)}{P^{J}4\left(\left(2d^{2}+6d+4\right)f^{2}+\left(6d^{2}+18d+12\right)f+4d^{2}+12d+8\right)}+\frac{\beta \left(\alpha \left(-d-2\right)f-2d-4\right)\left(\left(b_{1}d+b_{1}\right)f+b_{1}d+b_{1}\right)\left({\omega_{1}}^{4}-{R_{1}}^{4}\right)}{P^{J}4\left(\left(2d^{2}+6d+4\right)f^{2}+\left(6d^{2}+18d+12\right)f+4d^{2}+12d+8\right)}+\frac{\left(\left(-d-1\right)f-d-1\right)\left(\left({2a}_{1}d+4a_{1}\right)f+4a_{1}d+8a_{1}\right)\alpha \left({\omega_{1}}^{3}-{R_{1}}^{3}\right)}{P^{J}3\left(\left(2d^{2}+6d+4\right)f^{2}+\left(6d^{2}+18d+12\right)f+4d^{2}+12d+8\right)}+\frac{\alpha \left(\left({2a}_{1}d+4a_{1}\right)f+4a_{1}d+8a_{1}\right)\left(\left(d+1\right)f\omega_{1}+d\omega_{1}+\omega_{1}-e\left(d+1\right){\omega_{1}}^{f+1}-\left(m\left(\xi \right)-1\right)c{\omega_{1}}^{d+1}\left(f+1\right)\right)\left({\omega_{1}}^{2}-{R_{1}}^{2}\right)}{P^{J}2\left(\left(2d^{2}+6d+4\right)f^{2}+\left(6d^{2}+18d+12\right)f+4d^{2}+12d+8\right)}+\frac{\alpha \left(\left(b_{1}d+b_{1}\right)f+b_{1}d+b_{1}\right)\left(-2e\left(d+2\right){\omega_{1}}^{f+2}-\left(\left(-d-2\right)f-2d-4\right){\omega_{1}}^{2}-c{\omega_{1}}^{d+2}\left(\left(2m\left(\xi \right)-2\right)f+4m\left(\xi \right)-4\right)\right)\left({\omega_{1}}^{2}-{R_{1}}^{2}\right)}{P^{J}2\left(\left(2d^{2}+6d+4\right)f^{2}+\left(6d^{2}+18d+12\right)f+4d^{2}+12d+8\right)}\right)-\frac{\left(a_{2}+b_{2}T\right)\left(-\left(d+1\right)\left(f+1\right)\omega_{1}+\left(d+1\right)\left(f+1\right)t_{1}+e\left(d+1\right){\omega_{1}}^{f+1}-e\left(d+1\right){t_{1}}^{f+1}\right)\left(2\alpha +\beta \left({\omega_{1}}^{2}-{R_{1}}^{2}\right)\right)\left({\omega_{1}}^{2}-{R_{1}}^{2}\right)}{P^{J}\left(d+1\right)\left(f+1\right)}-\frac{\left(a_{2}+b_{2}T\right)\left(\left(m\left(\xi \right)-1\right)c{\omega_{1}}^{d+1}\left(f+1\right)-\left(m\left(\xi \right)-1\right)c{t_{1}}^{d+1}\left(f+1\right)\right)\left(2\alpha +\beta \left({\omega_{1}}^{2}-{R_{1}}^{2}\right)\right)\left({\omega_{1}}^{2}-{R_{1}}^{2}\right)}{P^{J}\left(d+1\right)\left(f+1\right)}-\left(\frac{e\beta \left(a_{2}+b_{2}T\right)\left(d+1\right)\left({t_{1}}^{f+5}-{\omega_{1}}^{f+5}\right)}{P^{J}\left(f+5\right)\left(\left(d+1\right)f+d+1\right)}+\frac{e\alpha \left(a_{2}+b_{2}T\right)\left(d+1\right)\left({t_{1}}^{f+3}-{\omega_{1}}^{f+3}\right)}{P^{J}\left(f+3\right)\left(\left(d+1\right)f+d+1\right)}+\frac{\left(m\left(\xi \right)-1\right)c\beta \left(a_{2}+b_{2}T\right)\left(f+1\right)\left({t_{1}}^{d+5}-{\omega_{1}}^{d+5}\right)}{P^{J}\left(d+5\right)\left(\left(d+1\right)f+d+1\right)}+\frac{\left(m\left(\xi \right)-1\right)c\alpha \left(a_{2}+b_{2}T\right)\left(f+1\right)\left({t_{1}}^{d+3}-{\omega_{1}}^{d+3}\right)}{P^{J}\left(d+3\right)\left(\left(d+1\right)f+d+1\right)}+\frac{\beta \left(a_{2}+b_{2}T\right)\left(\left(-d-1\right)f-d-1\right)\left({t_{1}}^{5}-{\omega_{1}}^{5}\right)}{P^{J}5\left(\left(d+1\right)f+d+1\right)}+\frac{\beta \left(a_{2}+b_{2}T\right)\left(\left(d+1\right)ft_{1}+dt_{1}+t_{1}-e\left(d+1\right){t_{1}}^{f+1}-\left(m\left(\xi \right)-1\right)c{t_{1}}^{d+1}\left(f+1\right)\right)\left({t_{1}}^{4}-{\omega_{1}}^{4}\right)}{P^{J}4\left(\left(d+1\right)f+d+1\right)}+\frac{\alpha \left(a_{2}+b_{2}T\right)\left(\left(-d-1\right)f-d-1\right)\left({t_{1}}^{3}-{\omega_{1}}^{3}\right)}{P^{J}3\left(\left(d+1\right)f+d+1\right)}+\frac{\alpha \left(a_{2}+b_{2}T\right)\left(\left(d+1\right)ft_{1}+dt_{1}+t_{1}-e\left(d+1\right){t_{1}}^{f+1}-\left(m\left(\xi \right)-1\right)c{t_{1}}^{d+1}\left(f+1\right)\right)\left({t_{1}}^{2}-{\omega_{1}}^{2}\right)}{P^{J}2\left(\left(d+1\right)f+d+1\right)}\right)$$

Backorder Cost

(Equation A.9) $$T_{BB}=-g_{3}\left(\frac{e^{-\delta T}\left(a_{2}+b_{2}T\right)\left(e^{\delta \left(\omega_{2}-t_{1}\right)}-\delta e^{\delta t_{1}\left(\omega_{2}-t_{1}\right)}\right)}{\delta^{2}P^{J}}+\frac{\left(b_{3}\left(\delta \left(\left(T-\omega_{2}\right)-\omega_{2}\right)-1-a_{3}\delta -1\right)e^{\delta \left(\left(T-\omega_{2}\right)-\omega_{2}\right)-T}\right)}{\delta^{3}P^{J}}+\frac{\left(a_{2}+b_{2}T\right)\left(e^{\delta \left(t_{1}-T\right)}-e^{\delta }\left(\omega_{2}-T\right)\right)\left(T-\omega_{2}\right)}{\delta P^{J}}\right)$$

Cost of Lost Sales

(Equation A.10) $$T_{LL}=g_{4}\left(\frac{\left(a_{2}+b_{2}T\right)\left(\omega_{2}-t_{1}\right)}{P^{J}}-\frac{\left(a_{2}+b_{2}T\right)e^{\delta \left(\left(\omega_{2}-t_{1}\right)-T\right)}}{\delta P^{J}}+\frac{a_{3}\left(T-\omega_{2}\right)}{P^{J}}-\frac{b_{3}\left({T^{2}-\omega_{2}}^{2}\right)}{{2P}^{J}}+\frac{\left(b_{3}\left(\delta \left(T-\omega_{2}\right)-1\right)+\delta a_{3}\right)e^{\delta \left(\left(T-\omega_{2}\right)-T\right)}}{\delta^{2}P^{J}}\right)$$

### Case 3

Ameliorating Cost

(Equation A.11) $${AA}_{c}=g_{1}\;\left(\frac{\psi e\left(m\left(\xi \right)-1\right)cfR_{1}^{f+d}}{f+d}+\frac{\psi e^{2}R_{1}^{2}f}{2}+\psi eR_{1}^{f}-\left(\frac{2e^{2}\left(d+2\right)f\left(\left(b_{1}d+b_{1}\right)f+b_{1}d+b_{1}\right)\left({\omega_{1}}^{2f+2}-{R_{1}}^{2f+2}\right)}{P^{J}\left(2f+2\right)\left(\left(2d^{2}+6d+4\right)f^{2}+\left(6d^{2}+18d+12\right)f+4d^{2}+12d+8\right)}+\frac{e^{2}\left(d+1\right)f\left(\left({2a}_{1}d+4a_{1}\right)f+4a_{1}d+8a_{1}\right)\left({\omega_{1}}^{2f+1}-{R_{1}}^{2f+1}\right)}{P^{J}\left(2f+1\right)\left(\left(2d^{2}+6d+4\right)f^{2}+\left(6d^{2}+18d+12\right)f+4d^{2}+12d+8\right)}+\frac{ecf\left(\left(2m\left(\xi \right)-2\right)f+4m\left(\xi \right)-4\right)\left(\left(b_{1}d+b_{1}\right)f+b_{1}d+b_{1}\right)\left({\omega_{1}}^{f+d+2}-{R_{1}}^{f+d+2}\right)}{P^{J}\left(f+d+2\right)\left(\left(2d^{2}+6d+4\right)f^{2}+\left(6d^{2}+18d+12\right)f+4d^{2}+12d+8\right)}+\frac{e\left(m\left(\xi \right)-1\right)cf\left(f+1\right)\left(\left({2a}_{1}d+4a_{1}\right)f+4a_{1}d+8a_{1}\right)\left({\omega_{1}}^{f+d+1}-{R_{1}}^{f+d+1}\right)}{P^{J}\left(f+d+1\right)\left(\left(2d^{2}+6d+4\right)f^{2}+\left(6d^{2}+18d+12\right)f+4d^{2}+12d+8\right)}+\frac{ef\left(\left(-d-2\right)f-2d-4\right)\left(\left(b_{1}d+b_{1}\right)f+b_{1}d+b_{1}\right)\left({\omega_{1}}^{f+2}-{R_{1}}^{f+2}\right)}{P^{J}\left(f+2\right)\left(\left(2d^{2}+6d+4\right)f^{2}+\left(6d^{2}+18d+12\right)f+4d^{2}+12d+8\right)}+\frac{ef\left(\left(-d-1\right)f-d-1\right)\left(\left({2a}_{1}d+4a_{1}\right)f+4a_{1}d+8a_{1}\right)\left({\omega_{1}}^{f+1}-{R_{1}}^{f+1}\right)}{P^{J}\left(f+1\right)\left(\left(2d^{2}+6d+4\right)f^{2}+\left(6d^{2}+18d+12\right)f+4d^{2}+12d+8\right)}+\frac{e\left(\left({2a}_{1}d+4a_{1}\right)f+4a_{1}d+8a_{1}\right)\left(\left(d+1\right)f\omega_{1}+d\omega_{1}+\omega_{1}-e\left(d+1\right){\omega_{1}}^{f+1}-\left(m\left(\xi \right)-1\right)c{\omega_{1}}^{d+1}\left(f+1\right)\right)\left({\omega_{1}}^{f}-{R_{1}}^{f}\right)}{P^{J}\left(\left(2d^{2}+6d+4\right)f^{2}+\left(6d^{2}+18d+12\right)f+4d^{2}+12d+8\right)}+\frac{e\left(\left(b_{1}d+b_{1}\right)f+b_{1}d+b_{1}\right)\left(-2e\left(d+2\right){\omega_{1}}^{f+2}-\left(\left(-d-2\right)f-2d-4\right){\omega_{1}}^{2}-c{\omega_{1}}^{d+2}\left(\left(2m\left(\xi \right)-2\right)f+4m\left(\xi \right)-4\right)\right)\left({\omega_{1}}^{f}-{R_{1}}^{f}\right)}{P^{J}\left(\left(2d^{2}+6d+4\right)f^{2}+\left(6d^{2}+18d+12\right)f+4d^{2}+12d+8\right)}\right)+\frac{e\left(a_{2}+b_{2}T\right)}{P^{J}}\left(\frac{\left(m\left(\xi \right)-1\right)c\left({\omega_{2}}^{d+1}-{\omega_{1}}^{d+1}\right)}{d+1}+\frac{e\left({\omega_{2}}^{f+1}-{\omega_{1}}^{f+1}\right)}{f+1}+\left({\omega_{2}}^{f}-{\omega_{1}}^{f}\right)\right)\left({\omega_{1}}^{f}-{R_{1}}^{f}\right)+\left(\frac{ea_{3}}{P^{J}}\left(\frac{\left(m\left(\xi \right)-1\right)c\left(t_{1}^{d+1}-{\omega_{2}}^{d+1}\right)}{d+1}+\frac{e\left(t_{1}^{f+1}-{\omega_{2}}^{f+1}\right)}{f+1}+\left(t_{1}-\omega_{2}\right)\right)+\frac{eb_{3}}{P^{J}}\left(\frac{\left(m\left(\xi \right)-1\right)c\left(t_{1}^{d+2}-{\omega_{2}}^{d+2}\right)}{d+2}-\frac{e\left(t_{1}^{f+2}-{\omega_{2}}^{f+2}\right)}{f+2}-\frac{\left({t_{1}}^{2}-{\omega_{2}}^{2}\right)}{2}\right)\left({\omega_{1}}^{f}-{R_{1}}^{f}\right)\right)-\left(\frac{e^{2}\left(a_{2}+b_{2}T\right)\left(d+1\right)f\left({\omega_{2}}^{2f+1}-{\omega_{1}}^{2f+1}\right)}{P^{J}\left(2f+1\right)\left(\left(d+1\right)f+d+1\right)}+\frac{e\left(m\left(\xi \right)-1\right)\left(a_{2}+b_{2}T\right)\left(f+1\right)cf\left({\omega_{2}}^{f+d+1}-{\omega_{1}}^{f+d+1}\right)}{P^{J}\left(f+d+1\right)\left(\left(d+1\right)f+d+1\right)}+\frac{e\left(a_{2}+b_{2}T\right)\left(\left(-d-1\right)f-d-1\right)f\left({\omega_{2}}^{f+1}-{\omega_{1}}^{f+1}\right)}{P^{J}\left(f+1\right)\left(\left(d+1\right)f+d+1\right)}+\frac{e\left(a_{2}+b_{2}T\right)\left(\left(d+1\right)f\omega_{2}+d\omega_{2}+\omega_{2}-e\left(d+1\right){\omega_{2}}^{f+1}-\left(m\left(\xi \right)-1\right)c{\omega_{2}}^{d+1}\left(f+1\right)\right)\left({\omega_{2}}^{f}-{\omega_{1}}^{f}\right)}{P^{J}\left(\left(d+1\right)f+d+1\right)}\right)+\left(\frac{ea_{3}}{P^{J}}\left(\frac{\left(m\left(\xi \right)-1\right)c\left(t_{1}^{d+1}-{\omega_{2}}^{d+1}\right)}{d+1}+\frac{e\left(t_{1}^{f+1}-{\omega_{2}}^{f+1}\right)}{f+1}+\left(t_{1}-\omega_{2}\right)\right)+\frac{eb_{3}}{P^{J}}\left(\frac{\left(m\left(\xi \right)-1\right)c\left(t_{1}^{d+2}-{\omega_{2}}^{d+2}\right)}{d+2}-\frac{e\left(t_{1}^{f+2}-{\omega_{2}}^{f+2}\right)}{f+2}-\frac{\left({t_{1}}^{2}-{\omega_{2}}^{2}\right)}{2}\right)\left({\omega_{2}}^{f}-{\omega_{1}}^{f}\right)\right)+\frac{2e^{2}\left(d+2\right)f\left(\left(b_{3}d+b_{3}\right)f+b_{3}d+b_{3}\right)\left({t_{1}}^{2f+2}-{\omega_{2}}^{2f+2}\right)}{P^{J}\left(2f+2\right)\left(\left(2d^{2}+6d+4\right)f^{2}+\left(6d^{2}+18d+12\right)f+4d^{2}+12d+8\right)}+\frac{e^{2}\left(d+1\right)f\left(\left({-2a}_{3}d-4a_{3}\right)f-4a_{3}d-8a_{3}\right)\left({t_{1}}^{2f+1}-{\omega_{2}}^{2f+1}\right)}{P^{J}\left(2f+1\right)\left(\left(2d^{2}+6d+4\right)f^{2}+\left(6d^{2}+18d+12\right)f+4d^{2}+12d+8\right)}+\frac{ecf\left(\left(2-2m\left(\xi \right)\right)f-4m\left(\xi \right)+4\right)\left(\left(b_{3}d+b_{3}\right)f+b_{3}d+b_{3}\right)\left({t_{1}}^{f+d+2}-{\omega_{2}}^{f+d+2}\right)}{P^{J}\left(f+d+2\right)\left(\left(2d^{2}+6d+4\right)f^{2}+\left(6d^{2}+18d+12\right)f+4d^{2}+12d+8\right)}+\frac{e\left(m\left(\xi \right)-1\right)cf\left(f+1\right)\left(\left({-2a}_{3}d-4a_{3}\right)f-4a_{3}d-8a_{3}\right)\left({t_{1}}^{f+d+1}-{\omega_{2}}^{f+d+1}\right)}{P^{J}\left(f+d+1\right)\left(\left(2d^{2}+6d+4\right)f^{2}+\left(6d^{2}+18d+12\right)f+4d^{2}+12d+8\right)}+\frac{ef\left(\left(d+2\right)f+2d+4\right)\left(\left(b_{3}d+b_{3}\right)f+b_{3}d+b_{3}\right)\left({t_{1}}^{f+2}-{\omega_{2}}^{f+2}\right)}{P^{J}\left(f+2\right)\left(\left(2d^{2}+6d+4\right)f^{2}+\left(6d^{2}+18d+12\right)f+4d^{2}+12d+8\right)}+\frac{ef\left(\left(d+1\right)f+d+1\right)\left(\left({-2a}_{3}d-4a_{3}\right)f-4a_{3}d-8a_{3}\right)\left({t_{1}}^{f+1}-{\omega_{2}}^{f+1}\right)}{P^{J}\left(f+1\right)\left(\left(2d^{2}+6d+4\right)f^{2}+\left(6d^{2}+18d+12\right)f+4d^{2}+12d+8\right)}+\frac{e\left(\left({-2a}_{3}d-4a_{3}\right)f-4a_{3}d-8a_{3}\right)\left(\left(-d-1\right)ft_{1}-dt_{1}-t_{1}-e\left(d+1\right){t_{1}}^{f+1}-\left(m\left(\xi \right)-1\right)c{t_{1}}^{d+1}\left(f+1\right)\right)\left({t_{1}}^{f}-{\omega_{2}}^{f}\right)}{P^{J}\left(\left(2d^{2}+6d+4\right)f^{2}+\left(6d^{2}+18d+12\right)f+4d^{2}+12d+8\right)}+\frac{e\left(\left(b_{3}d+b_{3}\right)f+b_{3}d+b_{3}\right)\left(-2e\left(d+2\right){t_{1}}^{f+2}-\left(\left(d+2\right)f+2d+4\right){t_{1}}^{2}-c{t_{1}}^{d+2}\left(\left(2-2m\left(\xi \right)\right)f-4m\left(\xi \right)+4\right)\right)\left({t_{1}}^{f}-{\omega_{2}}^{f}\right)}{P^{J}\left(\left(2d^{2}+6d+4\right)f^{2}+\left(6d^{2}+18d+12\right)f+4d^{2}+12d+8\right)}\right)$$

Deteriorating Cost

(Equation A.12) $${DD}_{c}=g_{2}\;\left(-\frac{\psi \left(m\left(\xi \right)-1\right)cR_{1}^{d}\left(2edR_{1}^{f}+\left(m\left(\xi \right)-1\right)c\left(f+d\right)R_{1}^{d}-2f-2d\right)fR_{1}^{f+d}}{2\left(f+d\right)}-\left(\frac{2e\left(1-m\left(\xi \right)\right)cd\left(d+2\right)\left(\left(b_{1}d+b_{1}\right)f+b_{1}d+b_{1}\right)\left({\omega_{1}}^{f+d+2}-{R_{1}}^{f+d+2}\right)}{P^{J}\left(f+d+2\right)\left(\left(2d^{2}+6d+4\right)f^{2}+\left(6d^{2}+18d+12\right)f+4d^{2}+12d+8\right)}+\frac{e\left(1-m\left(\xi \right)\right)cd\left(d+1\right)\left(\left({2a}_{1}d+4a_{1}\right)f+4a_{1}d+8a_{1}\right)\left({\omega_{1}}^{f+d+1}-{R_{1}}^{f+d+1}\right)}{P^{J}\left(f+d+1\right)\left(\left(2d^{2}+6d+4\right)f^{2}+\left(6d^{2}+18d+12\right)f+4d^{2}+12d+8\right)}+\frac{c^{2}\left(1-m\left(\xi \right)\right)d\left(\left(2m\left(\xi \right)-2\right)f+4m\left(\xi \right)-4\right)\left(\left(b_{1}d+b_{1}\right)f+b_{1}d+b_{1}\right)\left({\omega_{1}}^{2d+2}-{R_{1}}^{2d+2}\right)}{P^{J}\left(2d+2\right)\left(\left(2d^{2}+6d+4\right)f^{2}+\left(6d^{2}+18d+12\right)f+4d^{2}+12d+8\right)}+\frac{c^{2}\left(1-m\left(\xi \right)\right)\left(m\left(\xi \right)-1\right)d\left(f+1\right)\left(\left({2a}_{1}d+4a_{1}\right)f+4a_{1}d+8a_{1}\right)\left({\omega_{1}}^{2d+1}-{R_{1}}^{2d+1}\right)}{P^{J}\left(2d+1\right)\left(\left(2d^{2}+6d+4\right)f^{2}+\left(6d^{2}+18d+12\right)f+4d^{2}+12d+8\right)}+\frac{\left(1-m\left(\xi \right)\right)cd\left(\left(-d-2\right)f-2d-4\right)\left(\left(b_{1}d+b_{1}\right)f+b_{1}d+b_{1}\right)\left({\omega_{1}}^{d+2}-{R_{1}}^{d+2}\right)}{P^{J}\left(d+2\right)\left(\left(2d^{2}+6d+4\right)f^{2}+\left(6d^{2}+18d+12\right)f+4d^{2}+12d+8\right)}+\frac{\left(1-m\left(\xi \right)\right)cd\left(\left(-d-1\right)f-d-1\right)\left(\left({2a}_{1}d+4a_{1}\right)f+4a_{1}d+8a_{1}\right)\left({\omega_{1}}^{d+1}-{R_{1}}^{d+1}\right)}{P^{J}\left(d+1\right)\left(\left(2d^{2}+6d+4\right)f^{2}+\left(6d^{2}+18d+12\right)f+4d^{2}+12d+8\right)}+\frac{\left(1-m\left(\xi \right)\right)c\left(\left({2a}_{1}d+4a_{1}\right)f+4a_{1}d+8a_{1}\right)\left(\left(d+1\right)f\omega_{1}+d\omega_{1}+\omega_{1}-e\left(d+1\right){\omega_{1}}^{f+1}-\left(m\left(\xi \right)-1\right)c{\omega_{1}}^{d+1}\left(f+1\right)\right)\left({\omega_{1}}^{d}-{R_{1}}^{d}\right)}{P^{J}\left(\left(2d^{2}+6d+4\right)f^{2}+\left(6d^{2}+18d+12\right)f+4d^{2}+12d+8\right)}+\frac{\left(1-m\left(\xi \right)\right)c\left(\left(b_{1}d+b_{1}\right)f+b_{1}d+b_{1}\right)\left(-2e\left(d+2\right){\omega_{1}}^{f+2}-\left(\left(-d-2\right)f-2d-4\right){\omega_{1}}^{2}-c{\omega_{1}}^{d+2}\left(\left(2m\left(\xi \right)-2\right)f+4m\left(\xi \right)-4\right)\right)\left({\omega_{1}}^{d}-{R_{1}}^{d}\right)}{P^{J}\left(\left(2d^{2}+6d+4\right)f^{2}+\left(6d^{2}+18d+12\right)f+4d^{2}+12d+8\right)}\right)+\frac{\left(1-m\left(\xi \right)\right)c\left(a_{2}+b_{2}T\right)}{P^{J}}\left(\frac{\left(m\left(\xi \right)-1\right)c\left({\omega_{2}}^{d+1}-{\omega_{1}}^{d+1}\right)}{d+1}+\frac{e\left({\omega_{2}}^{f+1}-{\omega_{1}}^{f+1}\right)}{f+1}+\left({\omega_{2}}^{f}-{\omega_{1}}^{f}\right)\right)\left({\omega_{1}}^{d}-{R_{1}}^{d}\right)+\left(\frac{\left(1-m\left(\xi \right)\right)ca_{3}}{P^{J}}\left(\frac{\left(m\left(\xi \right)-1\right)c\left(t_{1}^{d+1}-{\omega_{2}}^{d+1}\right)}{d+1}+\frac{e\left(t_{1}^{f+1}-{\omega_{2}}^{f+1}\right)}{f+1}+\left(t_{1}-\omega_{2}\right)\right)+\frac{\left(1-m\left(\xi \right)\right)cb_{3}}{P^{J}}\left(\frac{\left(m\left(\xi \right)-1\right)c\left(t_{1}^{d+2}-{\omega_{2}}^{d+2}\right)}{d+2}-\frac{e\left(t_{1}^{f+2}-{\omega_{2}}^{f+2}\right)}{f+2}-\frac{\left({t_{1}}^{2}-{\omega_{2}}^{2}\right)}{2}\right)\left({\omega_{1}}^{d}-{R_{1}}^{d}\right)\right)-\left(\frac{c^{2}d\left(1-m\left(\xi \right)\right)\left(m\left(\xi \right)-1\right)\left(a_{2}+b_{2}T\right)\left(f+1\right)f\left({\omega_{2}}^{2d+1}-{\omega_{1}}^{2d+1}\right)}{P^{J}\left(2d+1\right)\left(\left(d+1\right)f+d+1\right)}+\frac{e\left(m\left(\xi \right)-1\right)\left(a_{2}+b_{2}T\right)\left(d+1\right)cd\left({\omega_{2}}^{f+d+1}-{\omega_{1}}^{f+d+1}\right)}{P^{J}\left(f+d+1\right)\left(\left(d+1\right)f+d+1\right)}+\frac{\left(1-m\left(\xi \right)\right)cd\left(a_{2}+b_{2}T\right)\left(\left(-d-1\right)f-d-1\right)\left({\omega_{2}}^{d+1}-{\omega_{1}}^{d+1}\right)}{P^{J}\left(d+1\right)\left(\left(d+1\right)f+d+1\right)}+\frac{\left(1-m\left(\xi \right)\right)c\left(a_{2}+b_{2}T\right)\left(\left(d+1\right)f\omega_{2}+d\omega_{2}+\omega_{2}-e\left(d+1\right){\omega_{2}}^{f+1}-\left(m\left(\xi \right)-1\right)c{\omega_{2}}^{d+1}\left(f+1\right)\right)\left({\omega_{2}}^{d}-{\omega_{1}}^{d}\right)}{P^{J}\left(\left(d+1\right)f+d+1\right)}\right)+\left(\frac{\left(1-m\left(\xi \right)\right)ca_{3}}{P^{J}}\left(\frac{\left(m\left(\xi \right)-1\right)c\left(t_{1}^{d+1}-{\omega_{2}}^{d+1}\right)}{d+1}+\frac{e\left(t_{1}^{f+1}-{\omega_{2}}^{f+1}\right)}{f+1}+\left(t_{1}-\omega_{2}\right)\right)+\frac{\left(1-m\left(\xi \right)\right)cb_{3}}{P^{J}}\left(\frac{\left(m\left(\xi \right)-1\right)c\left(t_{1}^{d+2}-{\omega_{2}}^{d+2}\right)}{d+2}-\frac{e\left(t_{1}^{f+2}-{\omega_{2}}^{f+2}\right)}{f+2}-\frac{\left({t_{1}}^{2}-{\omega_{2}}^{2}\right)}{2}\right)\left({\omega_{2}}^{d}-{\omega_{1}}^{d}\right)\right)+\frac{2e\left(1-m\left(\xi \right)\right)cd\left(d+2\right)\left(\left(b_{3}d+b_{3}\right)f+b_{3}d+b_{3}\right)\left({t_{1}}^{f+d+2}-{\omega_{2}}^{f+d+2}\right)}{P^{J}\left(f+d+2\right)\left(\left(2d^{2}+6d+4\right)f^{2}+\left(6d^{2}+18d+12\right)f+4d^{2}+12d+8\right)}+\frac{e\left(1-m\left(\xi \right)\right)cd\left(d+1\right)\left(\left({-2a}_{3}d-4a_{3}\right)f-4a_{3}d-8a_{3}\right)\left({t_{1}}^{f+d+1}-{\omega_{2}}^{f+d+1}\right)}{P^{J}\left(f+d+1\right)\left(\left(2d^{2}+6d+4\right)f^{2}+\left(6d^{2}+18d+12\right)f+4d^{2}+12d+8\right)}+\frac{c^{2}\left(1-m\left(\xi \right)\right)d\left(\left(2-2m\left(\xi \right)\right)f-4m\left(\xi \right)+4\right)\left(\left(b_{3}d+b_{3}\right)f+b_{3}d+b_{3}\right)\left({t_{1}}^{2d+2}-{\omega_{2}}^{2d+2}\right)}{P^{J}\left(2d+2\right)\left(\left(2d^{2}+6d+4\right)f^{2}+\left(6d^{2}+18d+12\right)f+4d^{2}+12d+8\right)}+\frac{c^{2}\left(1-m\left(\xi \right)\right)\left(m\left(\xi \right)-1\right)d\left(f+1\right)\left(\left({-2a}_{3}d-4a_{3}\right)f-4a_{3}d-8a_{3}\right)\left({t_{1}}^{2d+1}-{\omega_{2}}^{2d+1}\right)}{P^{J}\left(2d+1\right)\left(\left(2d^{2}+6d+4\right)f^{2}+\left(6d^{2}+18d+12\right)f+4d^{2}+12d+8\right)}+\frac{\left(1-m\left(\xi \right)\right)cd\left(\left(d+2\right)f+2d+4\right)\left(\left(b_{3}d+b_{3}\right)f+b_{3}d+b_{3}\right)\left({t_{1}}^{d+2}-{\omega_{2}}^{d+2}\right)}{P^{J}\left(d+2\right)\left(\left(2d^{2}+6d+4\right)f^{2}+\left(6d^{2}+18d+12\right)f+4d^{2}+12d+8\right)}+\frac{\left(1-m\left(\xi \right)\right)cd\left(\left(d+1\right)f+d+1\right)\left(\left({-2a}_{3}d-4a_{3}\right)f-4a_{3}d-8a_{3}\right)\left({t_{1}}^{d+1}-{\omega_{2}}^{d+1}\right)}{P^{J}\left(d+1\right)\left(\left(2d^{2}+6d+4\right)f^{2}+\left(6d^{2}+18d+12\right)f+4d^{2}+12d+8\right)}+\frac{\left(1-m\left(\xi \right)\right)c\left(\left({-2a}_{3}d-4a_{3}\right)f-4a_{3}d-8a_{3}\right)\left(\left(-d-1\right)ft_{1}-dt_{1}-t_{1}-e\left(d+1\right){t_{1}}^{f+1}-\left(m\left(\xi \right)-1\right)c{t_{1}}^{d+1}\left(f+1\right)\right)\left({t_{1}}^{d}-{\omega_{2}}^{d}\right)}{P^{J}\left(\left(2d^{2}+6d+4\right)f^{2}+\left(6d^{2}+18d+12\right)f+4d^{2}+12d+8\right)}+\frac{\left(1-m\left(\xi \right)\right)c\left(\left(b_{3}d+b_{3}\right)f+b_{3}d+b_{3}\right)\left(-2e\left(d+2\right){t_{1}}^{f+2}-\left(\left(d+2\right)f+2d+4\right){t_{1}}^{2}-c{t_{1}}^{d+2}\left(\left(2-2m\left(\xi \right)\right)f-4m\left(\xi \right)+4\right)\right)\left({t_{1}}^{d}-{\omega_{2}}^{d}\right)}{P^{J}\left(\left(2d^{2}+6d+4\right)f^{2}+\left(6d^{2}+18d+12\right)f+4d^{2}+12d+8\right)}\right)$$

Holding Cost

(Equation A.13) $${HH}_{c}=\frac{e\psi \beta R_{1}^{f+4}}{f+4}+\frac{e\psi \alpha R_{1}^{f+2}}{f+2}+\frac{\psi \left(m\left(\xi \right)-1\right)c\beta R_{1}^{d+4}}{d+4}+\frac{\psi \left(m\left(\xi \right)-1\right)c\alpha R_{1}^{d+2}}{d+2}+\frac{\psi \beta R_{1}^{4}+2\psi \alpha R_{1}^{2}}{d+2}-\left(\frac{2e\left(d+2\right)\left(\left(b_{1}d+b_{1}\right)f+b_{1}d+b_{1}\right)\beta \left({\omega_{1}}^{f+6}-{R_{1}}^{f+6}\right)}{P^{J}\left(f+6\right)\left(\left(2d^{2}+6d+4\right)f^{2}+\left(6d^{2}+18d+12\right)f+4d^{2}+12d+8\right)}+\frac{e\left(d+1\right)\left(\left({2a}_{1}d+4a_{1}\right)f+4a_{1}d+8a_{1}\right)\beta \left({\omega_{1}}^{f+5}-{R_{1}}^{f+5}\right)}{P^{J}\left(f+5\right)\left(\left(2d^{2}+6d+4\right)f^{2}+\left(6d^{2}+18d+12\right)f+4d^{2}+12d+8\right)}+\frac{2e\left(d+2\right)\left(\left(b_{1}d+b_{1}\right)f+b_{1}d+b_{1}\right)\alpha \left({\omega_{1}}^{f+4}-{R_{1}}^{f+4}\right)}{P^{J}\left(f+4\right)\left(\left(2d^{2}+6d+4\right)f^{2}+\left(6d^{2}+18d+12\right)f+4d^{2}+12d+8\right)}+\frac{e\left(d+1\right)\left(\left({2a}_{1}d+4a_{1}\right)f+4a_{1}d+8a_{1}\right)\alpha \left({\omega_{1}}^{f+3}-{R_{1}}^{f+3}\right)}{P^{J}\left(f+3\right)\left(\left(2d^{2}+6d+4\right)f^{2}+\left(6d^{2}+18d+12\right)f+4d^{2}+12d+8\right)}+\frac{c\left(\left(2m\left(\xi \right)-2\right)f+4m\left(\xi \right)-4\right)\left(\left(b_{1}d+b_{1}\right)f+b_{1}d+b_{1}\right)\beta \left({\omega_{1}}^{d+6}-{R_{1}}^{d+6}\right)}{P^{J}\left(d+6\right)\left(\left(2d^{2}+6d+4\right)f^{2}+\left(6d^{2}+18d+12\right)f+4d^{2}+12d+8\right)}+\frac{\left(m\left(\xi \right)-1\right)c\left(f+1\right)\left(\left({2a}_{1}d+4a_{1}\right)f+4a_{1}d+8a_{1}\right)\beta \left({\omega_{1}}^{d+5}-{R_{1}}^{d+5}\right)}{P^{J}\left(d+5\right)\left(\left(2d^{2}+6d+4\right)f^{2}+\left(6d^{2}+18d+12\right)f+4d^{2}+12d+8\right)}+\frac{c\left(\left(2m\left(\xi \right)-2\right)f+4m\left(\xi \right)-4\right)\left(\left(b_{1}d+b_{1}\right)f+b_{1}d+b_{1}\right)\alpha \left({\omega_{1}}^{d+4}-{R_{1}}^{d+4}\right)}{P^{J}\left(d+4\right)\left(\left(2d^{2}+6d+4\right)f^{2}+\left(6d^{2}+18d+12\right)f+4d^{2}+12d+8\right)}+\frac{\left(m\left(\xi \right)-1\right)c\left(f+1\right)\left(\left({2a}_{1}d+4a_{1}\right)f+4a_{1}d+8a_{1}\right)\alpha \left({\omega_{1}}^{d+3}-{R_{1}}^{d+3}\right)}{P^{J}\left(d+3\right)\left(\left(2d^{2}+6d+4\right)f^{2}+\left(6d^{2}+18d+12\right)f+4d^{2}+12d+8\right)}+\frac{\left(\left(-d-2\right)f-2d-4\right)\left(\left(b_{1}d+b_{1}\right)f+b_{1}d+b_{1}\right)\beta \left({\omega_{1}}^{6}-{R_{1}}^{6}\right)}{P^{J}6\left(\left(2d^{2}+6d+4\right)f^{2}+\left(6d^{2}+18d+12\right)f+4d^{2}+12d+8\right)}+\frac{\left(\left(-d-1\right)f-d-1\right)\left(\left({2a}_{1}d+4a_{1}\right)f+4a_{1}d+8a_{1}\right)\beta \left({\omega_{1}}^{5}-{R_{1}}^{5}\right)}{P^{J}5\left(\left(2d^{2}+6d+4\right)f^{2}+\left(6d^{2}+18d+12\right)f+4d^{2}+12d+8\right)}+\frac{\beta \left(\left({2a}_{1}d+4a_{1}\right)f+4a_{1}d+8a_{1}\right)\left(\left(d+1\right)f\omega_{1}+d\omega_{1}+\omega_{1}-e\left(d+1\right){\omega_{1}}^{f+1}-\left(m\left(\xi \right)-1\right)c{\omega_{1}}^{d+1}\left(f+1\right)\right)\left({\omega_{1}}^{4}-{R_{1}}^{4}\right)}{P^{J}4\left(\left(2d^{2}+6d+4\right)f^{2}+\left(6d^{2}+18d+12\right)f+4d^{2}+12d+8\right)}+\frac{\beta \left(\left(b_{1}d+b_{1}\right)f+b_{1}d+b_{1}\right)\left(-2e\left(d+2\right){\omega_{1}}^{f+2}-\left(\left(-d-2\right)f-2d-4\right){\omega_{1}}^{2}-c{\omega_{1}}^{d+2}\left(\left(2m\left(\xi \right)-2\right)f+4m\left(\xi \right)-4\right)\right)\left({\omega_{1}}^{4}-{R_{1}}^{4}\right)}{P^{J}4\left(\left(2d^{2}+6d+4\right)f^{2}+\left(6d^{2}+18d+12\right)f+4d^{2}+12d+8\right)}+\frac{\beta \left(\alpha \left(-d-2\right)f-2d-4\right)\left(\left(b_{1}d+b_{1}\right)f+b_{1}d+b_{1}\right)\left({\omega_{1}}^{4}-{R_{1}}^{4}\right)}{P^{J}4\left(\left(2d^{2}+6d+4\right)f^{2}+\left(6d^{2}+18d+12\right)f+4d^{2}+12d+8\right)}+\frac{\left(\left(-d-1\right)f-d-1\right)\left(\left({2a}_{1}d+4a_{1}\right)f+4a_{1}d+8a_{1}\right)\alpha \left({\omega_{1}}^{3}-{R_{1}}^{3}\right)}{P^{J}3\left(\left(2d^{2}+6d+4\right)f^{2}+\left(6d^{2}+18d+12\right)f+4d^{2}+12d+8\right)}+\frac{\alpha \left(\left({2a}_{1}d+4a_{1}\right)f+4a_{1}d+8a_{1}\right)\left(\left(d+1\right)f\omega_{1}+d\omega_{1}+\omega_{1}-e\left(d+1\right){\omega_{1}}^{f+1}-\left(m\left(\xi \right)-1\right)c{\omega_{1}}^{d+1}\left(f+1\right)\right)\left({\omega_{1}}^{2}-{R_{1}}^{2}\right)}{P^{J}2\left(\left(2d^{2}+6d+4\right)f^{2}+\left(6d^{2}+18d+12\right)f+4d^{2}+12d+8\right)}+\frac{\alpha \left(\left(b_{1}d+b_{1}\right)f+b_{1}d+b_{1}\right)\left(-2e\left(d+2\right){\omega_{1}}^{f+2}-\left(\left(-d-2\right)f-2d-4\right){\omega_{1}}^{2}-c{\omega_{1}}^{d+2}\left(\left(2m\left(\xi \right)-2\right)f+4m\left(\xi \right)-4\right)\right)\left({\omega_{1}}^{2}-{R_{1}}^{2}\right)}{P^{J}2\left(\left(2d^{2}+6d+4\right)f^{2}+\left(6d^{2}+18d+12\right)f+4d^{2}+12d+8\right)}\right)-\frac{\left(a_{2}+b_{2}T\right)\left(-\left(d+1\right)\left(f+1\right)\omega_{2}+\left(d+1\right)\left(f+1\right)\omega_{1}+e\left(d+1\right){\omega_{2}}^{f+1}-e\left(d+1\right){\omega_{1}}^{f+1}\right)\left(2\alpha +\beta t^{2}\right)\left({\omega_{1}}^{2}-{R_{1}}^{2}\right)}{P^{J}\left(d+1\right)\left(f+1\right)}-\frac{\left(a_{2}+b_{2}T\right)\left(\left(m\left(\xi \right)-1\right)c{\omega_{2}}^{d+1}\left(f+1\right)-\left(m\left(\xi \right)-1\right)c{\omega_{1}}^{d+1}\left(f+1\right)\right)\left(2\alpha +\beta t^{2}\right)\left({\omega_{1}}^{2}-{R_{1}}^{2}\right)}{P^{J}\left(d+1\right)\left(f+1\right)}+\left(\frac{\beta a_{3}}{4P^{J}}\left(\frac{\left(m\left(\xi \right)-1\right)c\left(t_{1}^{d+1}-{\omega_{2}}^{d+1}\right)}{d+1}+\frac{e\left(t_{1}^{f+1}-{\omega_{2}}^{f+1}\right)}{f+1}+\left(t_{1}-\omega_{2}\right)\right)+\frac{\beta b_{3}}{4P^{J}}\left(\frac{\left(m\left(\xi \right)-1\right)c\left(t_{1}^{d+2}-{\omega_{2}}^{d+2}\right)}{d+2}-\frac{e\left(t_{1}^{f+2}-{\omega_{2}}^{f+2}\right)}{f+2}-\frac{\left({t_{1}}^{2}-{\omega_{2}}^{2}\right)}{2}\right)\left({\omega_{1}}^{4}-{R_{1}}^{4}\right)\right)+\left(\frac{\alpha a_{3}}{2P^{J}}\left(\frac{\left(m\left(\xi \right)-1\right)c\left(t_{1}^{d+1}-{\omega_{2}}^{d+1}\right)}{d+1}+\frac{e\left(t_{1}^{f+1}-{\omega_{2}}^{f+1}\right)}{f+1}+\left(t_{1}-\omega_{2}\right)\right)+\frac{\alpha b_{3}}{2P^{J}}\left(\frac{\left(m\left(\xi \right)-1\right)c\left(t_{1}^{d+2}-{\omega_{2}}^{d+2}\right)}{d+2}-\frac{e\left(t_{1}^{f+2}-{\omega_{2}}^{f+2}\right)}{f+2}-\frac{\left({t_{1}}^{2}-{\omega_{2}}^{2}\right)}{2}\right)\left({\omega_{1}}^{2}-{R_{1}}^{2}\right)\right)-\left(\frac{e\beta \left(a_{2}+b_{2}T\right)\left(d+1\right)\left({\omega_{2}}^{f+5}-{\omega_{1}}^{f+5}\right)}{P^{J}\left(f+5\right)\left(\left(d+1\right)f+d+1\right)}+\frac{e\alpha \left(a_{2}+b_{2}T\right)\left(d+1\right)\left({\omega_{2}}^{f+3}-{\omega_{1}}^{f+3}\right)}{P^{J}\left(f+3\right)\left(\left(d+1\right)f+d+1\right)}+\frac{\left(m\left(\xi \right)-1\right)c\beta \left(a_{2}+b_{2}T\right)\left(f+1\right)\left({\omega_{2}}^{d+5}-{\omega_{1}}^{d+5}\right)}{P^{J}\left(d+5\right)\left(\left(d+1\right)f+d+1\right)}+\frac{\left(m\left(\xi \right)-1\right)c\alpha \left(a_{2}+b_{2}T\right)\left(f+1\right)\left({\omega_{2}}^{d+3}-{\omega_{1}}^{d+3}\right)}{P^{J}\left(d+3\right)\left(\left(d+1\right)f+d+1\right)}+\frac{\beta \left(a_{2}+b_{2}T\right)\left(\left(-d-1\right)f-d-1\right)\left({\omega_{2}}^{5}-{\omega_{1}}^{5}\right)}{P^{J}5\left(\left(d+1\right)f+d+1\right)}+\frac{\beta \left(a_{2}+b_{2}T\right)\left(\left(d+1\right)f\omega_{2}+d\omega_{2}+\omega_{2}-e\left(d+1\right){\omega_{2}}^{f+1}-\left(m\left(\xi \right)-1\right)c{\omega_{2}}^{d+1}\left(f+1\right)\right)\left({\omega_{2}}^{4}-{\omega_{1}}^{4}\right)}{P^{J}4\left(\left(d+1\right)f+d+1\right)}+\frac{\alpha \left(a_{2}+b_{2}T\right)\left(\left(-d-1\right)f-d-1\right)\left({\omega_{2}}^{3}-{\omega_{1}}^{3}\right)}{P^{J}3\left(\left(d+1\right)f+d+1\right)}+\frac{\alpha \left(a_{2}+b_{2}T\right)\left(\left(d+1\right)f\omega_{2}+d\omega_{2}+\omega_{2}-e\left(d+1\right){\omega_{2}}^{f+1}-\left(m\left(\xi \right)-1\right)c{\omega_{2}}^{d+1}\left(f+1\right)\right)\left({\omega_{2}}^{2}-{\omega_{1}}^{2}\right)}{P^{J}2\left(\left(d+1\right)f+d+1\right)}\right)+\left(\frac{\beta a_{3}}{4P^{J}}\left(\frac{\left(m\left(\xi \right)-1\right)c\left(t_{1}^{d+1}-{\omega_{2}}^{d+1}\right)}{d+1}+\frac{e\left(t_{1}^{f+1}-{\omega_{2}}^{f+1}\right)}{f+1}+\left(t_{1}-\omega_{2}\right)\right)+\frac{\beta b_{3}}{4P^{J}}\left(\frac{\left(m\left(\xi \right)-1\right)c\left(t_{1}^{d+2}-{\omega_{2}}^{d+2}\right)}{d+2}-\frac{e\left(t_{1}^{f+2}-{\omega_{2}}^{f+2}\right)}{f+2}-\frac{\left({t_{1}}^{2}-{\omega_{2}}^{2}\right)}{2}\right)\left({\omega_{2}}^{4}-{\omega_{1}}^{4}\right)\right)+\left(\frac{\alpha a_{3}}{2P^{J}}\left(\frac{\left(m\left(\xi \right)-1\right)c\left(t_{1}^{d+1}-{\omega_{2}}^{d+1}\right)}{d+1}+\frac{e\left(t_{1}^{f+1}-{\omega_{2}}^{f+1}\right)}{f+1}+\left(t_{1}-\omega_{2}\right)\right)+\frac{\alpha b_{3}}{2P^{J}}\left(\frac{\left(m\left(\xi \right)-1\right)c\left(t_{1}^{d+2}-{\omega_{2}}^{d+2}\right)}{d+2}+\frac{e\left(t_{1}^{f+2}-{\omega_{2}}^{f+2}\right)}{f+2}+\frac{\left({t_{1}}^{2}-{\omega_{2}}^{2}\right)}{2}\right)\left({\omega_{2}}^{2}-{\omega_{1}}^{2}\right)\right)+\frac{2e\left(d+2\right)\left(\left(b_{3}d+b_{3}\right)f+b_{3}d+b_{3}\right)\beta \left({t_{1}}^{f+6}-{\omega_{2}}^{f+6}\right)}{P^{J}\left(f+6\right)\left(\left(2d^{2}+6d+4\right)f^{2}+\left(6d^{2}+18d+12\right)f+4d^{2}+12d+8\right)}+\frac{e\left(d+1\right)\left(\left({-2a}_{3}d-4a_{3}\right)f-4a_{3}d-8a_{3}\right)\beta \left({t_{1}}^{f+5}-{\omega_{2}}^{f+5}\right)}{P^{J}\left(f+5\right)\left(\left(2d^{2}+6d+4\right)f^{2}+\left(6d^{2}+18d+12\right)f+4d^{2}+12d+8\right)}+\frac{2e\left(d+2\right)\left(\left(b_{3}d+b_{3}\right)f+b_{3}d+b_{3}\right)\alpha \left({t_{1}}^{f+4}-{\omega_{2}}^{f+4}\right)}{P^{J}\left(f+4\right)\left(\left(2d^{2}+6d+4\right)f^{2}+\left(6d^{2}+18d+12\right)f+4d^{2}+12d+8\right)}+\frac{e\left(d+1\right)\left(\left({-2a}_{3}d-4a_{3}\right)f-4a_{3}d-8a_{3}\right)\alpha \left({t_{1}}^{f+3}-{\omega_{2}}^{f+3}\right)}{P^{J}\left(f+3\right)\left(\left(2d^{2}+6d+4\right)f^{2}+\left(6d^{2}+18d+12\right)f+4d^{2}+12d+8\right)}+\frac{c\left(\left(2-2m\left(\xi \right)\right)f-4m\left(\xi \right)+4\right)\left(\left(b_{3}d+b_{3}\right)f+b_{3}d+b_{3}\right)\beta \left({t_{1}}^{d+6}-{\omega_{2}}^{d+6}\right)}{P^{J}\left(d+6\right)\left(\left(2d^{2}+6d+4\right)f^{2}+\left(6d^{2}+18d+12\right)f+4d^{2}+12d+8\right)}+\frac{\left(m\left(\xi \right)-1\right)c\left(f+1\right)\left(\left({-2a}_{3}d-4a_{3}\right)f-4a_{3}d-8a_{3}\right)\beta \left({t_{1}}^{d+5}-{\omega_{2}}^{d+5}\right)}{P^{J}\left(d+5\right)\left(\left(2d^{2}+6d+4\right)f^{2}+\left(6d^{2}+18d+12\right)f+4d^{2}+12d+8\right)}+\frac{c\left(\left(2-2m\left(\xi \right)\right)f-4m\left(\xi \right)+4\right)\left(\left(b_{3}d+b_{3}\right)f+b_{3}d+b_{3}\right)\alpha \left({t_{1}}^{d+4}-{\omega_{2}}^{d+4}\right)}{P^{J}\left(d+4\right)\left(\left(2d^{2}+6d+4\right)f^{2}+\left(6d^{2}+18d+12\right)f+4d^{2}+12d+8\right)}+\frac{\left(m\left(\xi \right)-1\right)c\left(f+1\right)\left(\left({-2a}_{3}d-4a_{3}\right)f-4a_{3}d-8a_{3}\right)\alpha \left({t_{1}}^{d+3}-{\omega_{2}}^{d+3}\right)}{P^{J}\left(d+3\right)\left(\left(2d^{2}+6d+4\right)f^{2}+\left(6d^{2}+18d+12\right)f+4d^{2}+12d+8\right)}+\frac{\left(\left(d+2\right)f+2d+4\right)\left(\left(b_{3}d+b_{3}\right)f+b_{3}d+b_{3}\right)\beta \left({t_{1}}^{6}-{\omega_{2}}^{6}\right)}{P^{J}6\left(\left(2d^{2}+6d+4\right)f^{2}+\left(6d^{2}+18d+12\right)f+4d^{2}+12d+8\right)}+\frac{\left(\left(d+1\right)f+d+1\right)\left(\left({-2a}_{3}d-4a_{3}\right)f-4a_{3}d-8a_{3}\right)\beta \left({t_{1}}^{5}-{\omega_{2}}^{5}\right)}{P^{J}5\left(\left(2d^{2}+6d+4\right)f^{2}+\left(6d^{2}+18d+12\right)f+4d^{2}+12d+8\right)}+\frac{\beta \left(\left({-2a}_{3}d-4a_{3}\right)f-4a_{3}d-8a_{3}\right)\left(\left(-d-1\right)ft_{1}-dt_{1}-t_{1}-e\left(d+1\right){t_{1}}^{f+1}-\left(m\left(\xi \right)-1\right)c{t_{1}}^{d+1}\left(f+1\right)\right)\left({t_{1}}^{4}-{\omega_{2}}^{4}\right)}{P^{J}4\left(\left(2d^{2}+6d+4\right)f^{2}+\left(6d^{2}+18d+12\right)f+4d^{2}+12d+8\right)}+\frac{\beta \left(\left(b_{3}d+b_{3}\right)f+b_{3}d+b_{3}\right)\left(-2e\left(d+2\right){t_{1}}^{f+2}-\left(\left(d+2\right)f+2d+4\right){t_{1}}^{2}-c{t_{1}}^{d+2}\left(\left(2-2m\left(\xi \right)\right)f-4m\left(\xi \right)+4\right)\right)\left({t_{1}}^{4}-{\omega_{2}}^{4}\right)}{P^{J}4\left(\left(2d^{2}+6d+4\right)f^{2}+\left(6d^{2}+18d+12\right)f+4d^{2}+12d+8\right)}+\frac{\beta \left(\alpha \left(d+2\right)f+2d+4\right)\left(\left(b_{3}d+b_{3}\right)f+b_{3}d+b_{3}\right)\left({t_{1}}^{4}-{\omega_{2}}^{4}\right)}{P^{J}4\left(\left(2d^{2}+6d+4\right)f^{2}+\left(6d^{2}+18d+12\right)f+4d^{2}+12d+8\right)}+\frac{\left(\left(d+1\right)f+d+1\right)\left(\left({-2a}_{3}d-4a_{3}\right)f-4a_{3}d-8a_{3}\right)\alpha \left({t_{1}}^{3}-{\omega_{2}}^{3}\right)}{P^{J}3\left(\left(2d^{2}+6d+4\right)f^{2}+\left(6d^{2}+18d+12\right)f+4d^{2}+12d+8\right)}+\frac{\alpha \left(\left({-2a}_{3}d-4a_{3}\right)f-4a_{3}d-8a_{3}\right)\left(\left(-d-1\right)ft_{1}-dt_{1}-t_{1}-e\left(d+1\right){t_{1}}^{f+1}-\left(m\left(\xi \right)-1\right)c{t_{1}}^{d+1}\left(f+1\right)\right)\left({t_{1}}^{2}-{\omega_{2}}^{2}\right)}{P^{J}2\left(\left(2d^{2}+6d+4\right)f^{2}+\left(6d^{2}+18d+12\right)f+4d^{2}+12d+8\right)}+\frac{\alpha \left(\left(b_{3}d+b_{3}\right)f+b_{3}d+b_{3}\right)\left(-2e\left(d+2\right){t_{1}}^{f+2}-\left(\left(d+2\right)f+2d+4\right){t_{1}}^{2}-c{t_{1}}^{d+2}\left(\left(2-2m\left(\xi \right)\right)f-4m\left(\xi \right)+4\right)\right)\left({t_{1}}^{2}-{\omega_{2}}^{2}\right)}{P^{J}2\left(\left(2d^{2}+6d+4\right)f^{2}+\left(6d^{2}+18d+12\right)f+4d^{2}+12d+8\right)}$$

Backorder Cost

(Equation A.14) $$T_{BB}=-g_{3}\left(\frac{\left(b_{3}\left(\delta \left(\left(T-t_{1}\right)-t_{1}\right)-1-a_{3}\delta -1\right)e^{\delta \left(\left(T-t_{1}\right)-t_{1}\right)-T}\right)}{\delta^{3}P^{J}}\right)$$

Cost of Lost Sales

(Equation A.15) $$T_{LL}=g_{4}\left(\frac{a_{3}\left(T-t_{1}\right)}{P^{J}}-\frac{b_{3}\left({T^{2}-t_{1}}^{2}\right)}{{2P}^{J}}+\frac{\left(b_{3}\left(\delta \left(T-t_{1}\right)-1\right)+\delta a_{3}\right)e^{\delta \left(\left(T-t_{1}\right)-T\right)}}{\delta^{2}P^{J}}\right)$$
